# Identification of herpesvirus transcripts from genomic regions around the replication origins

**DOI:** 10.1038/s41598-023-43344-y

**Published:** 2023-09-29

**Authors:** Gábor Torma, Dóra Tombácz, Zsolt Csabai, Islam A. A. Almsarrhad, Gergely Ármin Nagy, Balázs Kakuk, Gábor Gulyás, Lauren McKenzie Spires, Ishaan Gupta, Ádám Fülöp, Ákos Dörmő, István Prazsák, Máté Mizik, Virág Éva Dani, Viktor Csányi, Ákos Harangozó, Zoltán Zádori, Zsolt Toth, Zsolt Boldogkői

**Affiliations:** 1 Department of Medical Biology, Albert Szent-Györgyi Medical School, University of Szeged, Szeged, Hungary; 2 MTA -SZTE Lendület GeMiNI Research Group, University of Szeged, Szeged, Hungary; 3 Department of Oral Biology, University of Florida College of Dentistry, Gainesville, FL USA; 4 Department of Biochemical Engineering and Biotechnology, Indian Institute of Technology, Delhi, India; 5 HUN-REN Veterinary Medical Research Institute HU, Budapest, Hungary

**Keywords:** Herpes virus, Transcriptomics

## Abstract

Long-read sequencing (LRS) techniques enable the identification of full-length RNA molecules in a single run eliminating the need for additional assembly steps. LRS research has exposed unanticipated transcriptomic complexity in various organisms, including viruses. Herpesviruses are known to produce a range of transcripts, either close to or overlapping replication origins (Oris) and neighboring genes related to transcription or replication, which possess confirmed or potential regulatory roles. In our research, we employed both new and previously published LRS and short-read sequencing datasets to uncover additional Ori-proximal transcripts in nine herpesviruses from all three subfamilies (alpha, beta and gamma). We discovered novel long non-coding RNAs, as well as splice and length isoforms of mRNAs. Moreover, our analysis uncovered an intricate network of transcriptional overlaps within the examined genomic regions. We demonstrated that herpesviruses display distinct patterns of transcriptional overlaps in the vicinity of or at the Oris. Our findings suggest the existence of a ‘super regulatory center’ in the genome of alphaherpesviruses that governs the initiation of both DNA replication and global transcription through multilayered interactions among the molecular machineries.

## Introduction

### Regulation of herpesvirus transcription

The lytic transcription of herpesviruses follows a sequential order, which is divided into three distinct temporal phases: immediate-early (IE), early (E), and late (L)^1^. The IE gene products activate the transcription of both E genes, which encode enzymes of DNA replication, and L genes that produce structural proteins. Four IE proteins participate in the transcription regulation of herpes simplex virus type 1 (HSV-1), a representative member of alphaherpesviruses (αHVs). The essential ICP4 viral protein, encoded by *rs1* (*icp4*) serves as the main transcription regulator (TR), recruiting cellular transcription factors (e.g. TFIID) to viral promoters to enhance transcription initiation^2^. ICP22, encoded by *us1* promotes the transcription elongation^3^. The *us1* gene of HSV-1 is located in the unique short (US) genomic region in a single copy, while its promoter is represented in duplicate in the inverted repeat (IR) region (the other IR copy controls the expression of *ul12* gene). In the Varicellovirus genus, the *us1* gene is translocated to the IR region making it present in duplicate. ICP0, encoded by *rl2* (*icp0*)], is not a transcription factor in the strict sense, but it can promote transcription by affecting pre-chromatin interactions before histones bind to viral DNA^4^. ICP27, encoded by *ul54* participates in recruiting RNA polymerase (RNP) to viral promoters^5^ and DNA synthesis^6^.

The mechanism controlling the lytic cycle in other αHVs is comparable to that of HSV-1. One significant difference is the expression kinetics of *icp0*, *us1* and *ul54* orthologs, which have evolved to be expressed in the E kinetic phase in pseudorabies virus (PRV) and equid alphaherpesvirus type 1 (EHV-1). Additionally, the *icp0* gene has been structurally and functionally simplified in these viruses. Betaherpesviruses (βHVs) and gammaherpesviruses (γHVs) employ similar mechanisms as αHVs for controlling genome-wide viral transcription. In human cytomegalovirus (HCMV), the prototype member of βHVs, two major IE genes (*ie1* and *ie2*) regulate global viral transcription^7^. The IE proteins of Epstein-Barr virus (EBV; the representative member of γHVs), designated as BZLF1 and BRLF1, are transactivators that initiate the transcription of E viral genes^8^.

An alternative way herpesviruses operate is by establishing latency, during which the majority of the viral genome remains transcriptionally silent, with only a few specific viral RNAs being expressed. The latency-associated transcript (LAT) of HSV-1 is the sole viral gene product that is highly expressed during the latent state^9^. This non-coding RNA (ncRNA) represses the lytic gene expression through blocking the activity of *icp4*^10^ and facilitating heterochromatin formation on the HSV-1 genome^11^. Although LAT contains numerous open reading frames (ORFs), it likely does not encode any polypeptides^12,13^. Other long ncRNAs (lncRNAs) are also expressed during latency, such as the long-latency transcript (LLT; overlapping *icp0* and *icp4* genes^14^) and the L/S junction-spanning transcripts (L/STs; overlapping *icp34.5* and *icp4* genes^15^).

Members of the non-coding NOIR-1 transcript family, described in αHVs [PRV^16^, varicella-zoster virus (VZV)^17^, and EHV-1^18^] are 3′-coterminal with the LLT transcripts and are expressed during the lytic cycle. The low-abundance NOIR-2 RNA, which is transcribed in a reverse orientation relative to NOIR-1, has only been detected in PRV^16^. ELIE, another lytic lncRNA of PRV^19^, partially overlaps the long isoform of HSV-1 L/ST.

### DNA replication

While the prokaryotic genomes contain a single start site of DNA synthesis (designated as replication origin or Ori) defined by consensus sequences^20^, eukaryotic genomes typically have tens of thousands Oris specified by their chromatin structure^21,22^. Viruses have a single or a few Oris, which are specified by a combination of structural properties and sequence specificity of the particular DNA segment^23^. The replication of eukaryotic genomes is initiated by the binding of the origin recognition complex (ORC) to the Ori^24^. The function of ORC is to serve as a platform for the assembly of the replisome, which is composed of a wide range of proteins such as DNA helicase, DNA polymerase (DNP), topoisomerase, primase, DNA gyrase, single-stranded DNA-binding protein (ssDBP), RNase H, DNA ligase, and telomerase enzymes.

Herpesviruses encode several replication proteins needed for DNA synthesis. For example, HSV-1 codes for an origin-binding protein (OBP) (*ul9*), an ssDBP (*ul29*), two DNPs (*ul30* and *ul42*), and three helicase/primase molecules (*ul5*, *ul8*, and *ul52*)^25,26^. Several viral auxiliary factors play roles in the nucleotide metabolism, such as ribonucleotide reductase (*ul39/40*), thymidine kinase (*ul23*), uracil-DNA glycosylase (*ul2*), deoxyuridine triphosphatase (*ul50*), alkaline nuclease (*ul12*), allowing herpesvirus replication in non-dividing cells^27^.

HSV-1 contains three Oris, two within the IRs surrounding the US region (termed OriS), and one in the unique long (UL) region (termed OriL) (Supplementary Fig. 1). In Simplexviruses, OriL is located between two E genes, *ul29* and *ul30*, involved in DNA replication, while the two copies of OriS are surrounded by the IE genes, *icp4* and *us1*, within the internal repeat of the US region (IRS), and by *icp4* and *us12* genes within the terminal repeat of the US region (TRS). Some members of Varicelloviruses, such as VZV and Bovine alphaherpesvirus 1 (BoHV-1) lack an OriL. However, in other Varicelloviruses like PRV and EHV-1, the position of OriL has shifted to the intergenic region between the *ul21* and *ul22* gene pair. HCMV has only a single replication origin (OriLyt), located at a semi-orthologous position, next to *ul57* homologous to the HSV-1 *ul29*. Human gammaherpesviruses, EBV and Kaposi’s sarcoma herpesvirus (KSHV), have two lytic (OriLyt-L and OriLyt-R), and a latent replication origin (termed OriP in EBV and terminal repeat in KSHV)^28–31^. The EBV OriP comprises two main components: the dyad symmetry (DS) and the family of repeats (FR). Each of them hosts multiple binding sites for the protein EBNA-1. When EBNA1 binds to the DS, it functions as an Ori, facilitating the recruitment of ORC^32^. DNA replication in its lytic phase relies on seven EBV replication proteins. BZLF1 binds to the OriLyt. It has the ability to bind multiple viral replication proteins and thereby initiating the lytic phase. The BALF5 protein encodes the catalytic subunit of DNP and interacts with the helicase-primase complex. BALF2 acts as the ssDBP. The BMRF1 protein is a DNP accessory subunit, which can serve as a coactivator of BZLF1 and it interacts with BALF5, forming the DNP holoenzyme^33^.

### Overlapping viral transcripts

Recent studies^34^ have shown that every herpesvirus gene forms various transcriptional overlaps (TOs), including divergent (head-to-head), convergent (tail-to-tail), and parallel (tail-to-head). Tandem genes form parallel-overlapping multigenic, 3´-coterminal transcripts, representing the archetypal genomic organization of herpesviruses. Moreover, many viral genes express 5´-truncated transcripts with different transcription start sites (TSSs) but the same transcription end site (TES), containing ‘nested’ open reading frames (ORFs) which encode N-terminally-truncated polypeptides^35,36^. Most co-located divergent genes produce ‘hard’ TOs where the canonical transcripts overlap each other. However, in a few cases, only the long transcript isoforms (TIs), create head-to head TOs (‘soft’ TOs), not the canonical transcript. Convergently oriented genes form ‘soft’ TOs through transcriptional read-through, but only a few ‘hard’ TOs can be observed (e.g. in αHVs, the *ul7/8*, *ul30/31*, and *ul50/51* gene pairs^37^).

### Non-coding RNAs regulating DNA replication

There is growing evidence that certain non-coding transcripts, including short ncRNAs, such as microRNAs (miRNAs^38^), and lncRNAs, play essential roles in regulating DNA replication^39^. For instance, a specific type of lncRNAs encoded by sequences near the Oris has been found in all three domains of life and in viruses over the past decade. A recent survey revealed that approximately 72% of mammalian ORC1s are associated with active promoters, more than half of them controlled by ncRNAs^40^. Replication RNAs have several modes for controlling DNA replication. These include the regulation of RNA primer synthesis through hybridization with DNA sequences^9^, or the formation of hybrids with mRNAs. This latter process initiates their degradation by RNase H, thereby inhibiting the translation of replication proteins. Additionally, these transcripts can help recruit ORC to the Ori^41^.

### Replication origin-associated herpesvirus transcripts

Replication origin-associated RNAs (raRNAs) have been identified in all three subfamilies of herpesviruses. While many of these transcripts have previously been described in βHVs and γHVs, they were practically undetected in αHVs until recently. For example, the non-coding RNA4.9 of HCMV, transcribed from the OriLyt, has been identified^42^ and functionally characterized^43^. This transcript regulates viral DNA replication through the formation of a DNA:RNA hybrid and also affects the expression level of ssDBP encoded by the *ul57* gene. RNA4.9 is thought to have additional roles, operating in both *cis* and *trans*, such as promoting transcriptional repression of the major IE promoter during latency^44^. The discovery of RNA4.9 and other HCMV raRNAs led to the belief that this virus has a unique mode of replication regulation, which has not proven to be true. Other raRNAs (SRT, vRNA-1 and vRNA-2) overlapping the OriLyt have also been described in HCMV^45,46^. The formation of an RNA:DNA hybrid by the BHLF1 replication RNA at the EBV OriLyt region has been described earlier as well^47^. Additionally, a bidirectional promoter^48^ and a highly structured RNA were identified within this region of EBV^44^. The function of this latter transcript is to assist the viral EBNA1 and HMGA1a proteins in recruiting ORC^49^. Incomplete sequences of two co-terminal lncRNAs near the HSV-1 OriS have been described previously^50^.

The emergence of long-read sequencing (LRS) technologies has greatly accelerated the discovery of novel viral transcripts and their TIs, including splice, TSS and TES variants. These investigations have identified numerous lncRNAs near both the OriS and OriL regions of αHVs^34,51–53^. However, since no precise function has been linked to these transcripts, it would be inaccurate to term them as ‘replication RNAs’ as their role in DNA replication is still undisclosed. Moreover, genes surrounding the Oris have been shown to produce TIs with long 5′-untranslated regions (5′ UTRs—TSS isoforms), or 3′ UTRs (TES isoforms) that overlap the replication origin^54,55^.

## Results

### Multiplatform sequencing for characterization of herpesvirus transcripts

**Figure 1:**
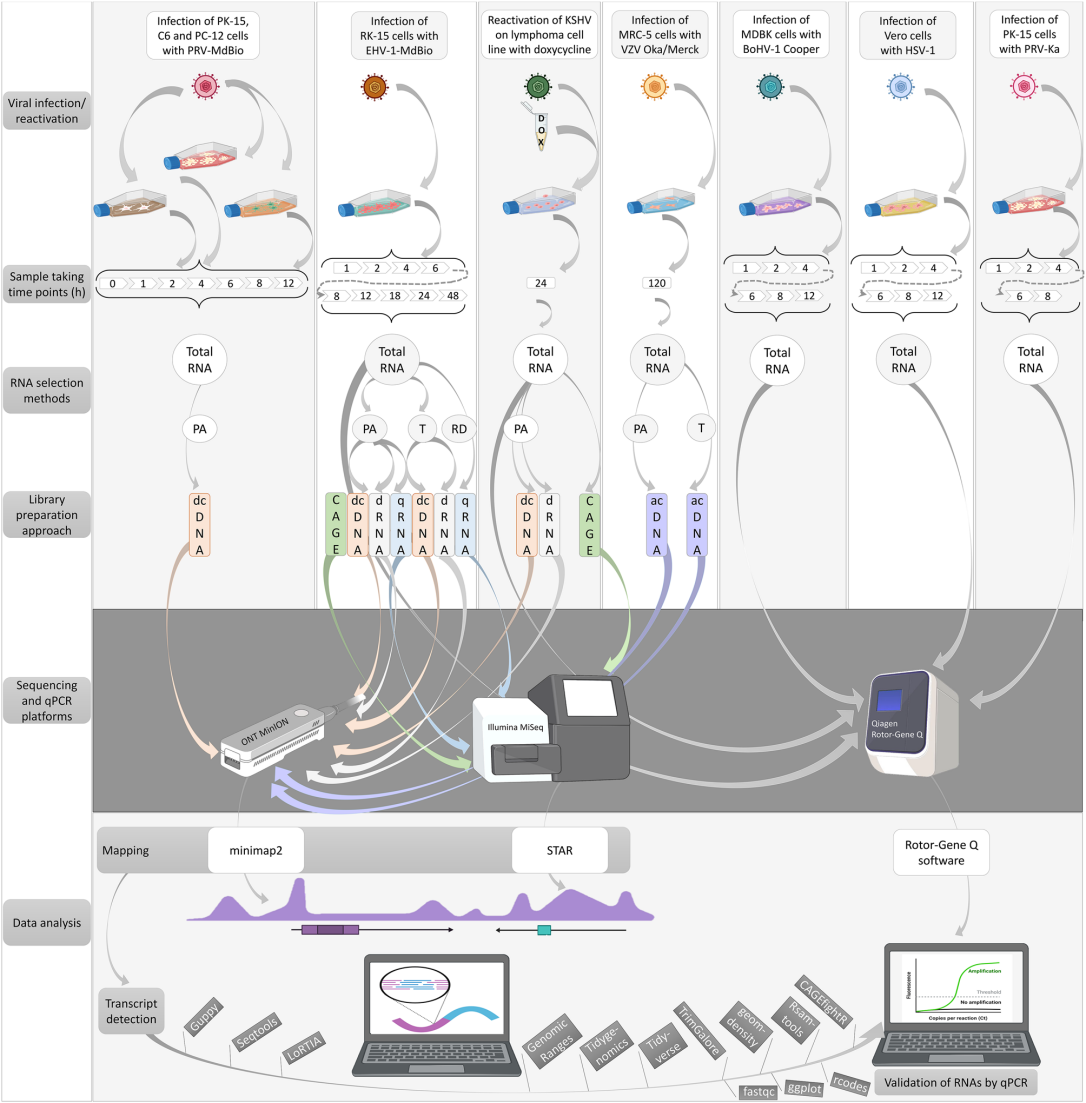
Workflow. Here, we outline the techniques employed to generate new sequencing data, including infection of cells with various viruses, library preparation, sequencing and bioinformatics. The qRT-PCR validation workflow for several transcripts is also depicted. Abbreviations: PA: polyadenylated RNAs; T: Terminator-handled samples; RD: ribodepleted RNAs; dcDNA: direct cDNA-seq; CAGE: Cap Analysis Gene Expression-Seq; dRNA: direct RNA-Seq; qRNA: short-read RNA-Seq (library generated by qRNA-seq kit); acDNA: amplified cDNA-Seq.

In this study, we applied newly generated (Fig. 1) and previously published sequencing data (Table 1) for analyzing the Ori-proximal transcripts of nine herpesviruses. The following approaches were used for data production: SRS on different Illumina platforms^51,56–59^, as well as LRS on ONT (MinION^18^), Pacific Biosciences (PacBio—RSII and Sequel^16,60^), and LoopSeq^53^ platforms along with a wide range of library preparation techniques and Cap Analysis of Gene Expression sequencing (CAGE-Seq, conducted on an Illumina platform) for VZV^61^ and EBV^60^. The terminology for orthologous genes varies among the αHVs. To enhance comparability, we have adopted the naming convention used for HSV-1(Supplementary Table 1). Our procedure for annotating and verifying transcripts was as follows. We first identified a sequencing read obtained by direct cDNA sequencing (dcDNA-Seq) across all three biological replicates. The TSS and TES of this read were identified by CAGE-Seq, RAMPAGE, and direct RNA sequencing (dRNA-Seq). Additionally, we required that the transcript itself be detectable by dRNA-Seq and quantitative reverse transcription PCR (qRT-PCR). For intron annotation, their detection via dRNA-Seq was necessary. However, as we also relied on previously published data, we could not employ all these methods in every virus and dataset. In the respective figures, we indicated which techniques were utilized for the detection and validation of the given transcripts.

**Table 1:** Techniques and datasets from earlier publications used in this study.

Virus	Sequencing approach	Library	References	Data availability
*BoHV-1*	LRS ONT	direct RNA	^53^	ENA: PRJEB33511
	LRS ONT	direct cDNA		
	LRS ONT	amplified cDNA		
	Synthetic LRS Illumina	LoopSeq		
	LRS ONT	direct cDNA	^74^	ENA: PRJEB33511
*EBV*	LRS PacBio RSII	amplified cDNA	^60,77^	GSE: GSE79337
	SRS Illumina	amplified cDNA		
		CAGE		
	LRS ONT	direct cDNA	^84^	ENA: PRJEB38992
		amplified cDNA		
*HCMV*	LRS PacBio RSII	amplified cDNA	^78^	ENA: PRJEB22072
	LRS PacBio Sequel	amplified cDNA	^83^	ENA: PRJEB25680
	LRS ONT	amplified cDNA		
	LRS ONT	direct RNA		
	LRS ONT	CAP-selected	^36^	ENA: PRJEB25680
*HSV-1*	SRS Illumina	amplified cDNA	^56^	GEO: GSE59717
		amplified cDNA	^57^	SRA: PRJNA505045
		amplified cDNA	^58^	SRA: PRJNA482043, PRJNA483305, and PRJNA533478
		amplified cDNA	^59^	GEO: GSE128324
	LRS PacBio Sequel	amplified cDNA	^79^	ENA: PRJEB25433
	LRS ONT	amplified cDNA		
		direct RNA		
	LRS ONT	direct RNA	^80^	ENA: PRJEB27861
	LRS PacBio RSII	amplified cDNA	^37^	GEO: GSE97785
*PRV*	SRS Illumina	amplified cDNA	^62^	ENA: PRJEB9526
	LRS PacBio RSII	direct cDNA	^16^	ENA: PRJEB12867
		amplified cDNA		
	LRS PacBio Sequel	amplified cDNA	^37^	ENA: PRJEB24593
	LRS ONT	direct RNA		
	LRS ONT	amplified cDNA		
	LRS ONT	amplified Cap-selected		
	LRS ONT	Terminator-handled amplified cDNA	^55^	ENA: ERP106430 and ERP019579
	LRS ONT	direct cDNA		
	LRS ONT	direct RNA		
*SVV*	LRS ONT	direct RNA	^61^	ENA: PRJEB42868
	SRS Illumina	amplified cDNA		
*VZV*	LRS ONT	amplified cDNA	^17^	ENA: PRJEB25401
		Targeted		
	LRS ONT	amplified Cap-selected	^81^	ENA: PRJEB25401
	SRS Illumina	amplified cDNA	^75^	ENA: PRJEB38829
	SRS Illumina	CAGE		
	LRS ONT	direct RNA		

This table show the annotated transcripts mapping to the genomic loci examined in this study.

In the first part of this study, we precisely annotated the genomic locations of canonical transcripts and their TIs, including TSSs, TESs, and splice variants. Previous annotations were either verified, or in several instances, revised based on the integration and reassessment of sequencing datasets. We also identified cis-regulatory elements for numerous RNA molecules. Notably, we identified promoter elements (TATA boxes) within the Ori regions in all cases. Our objective was to offer a comprehensive view of the complexity of TOs in the investigated genomic regions. In viruses lacking either dRNA-Seq or CAGE data, we employed stricter criteria for transcript annotation, resulting in lower transcriptomic complexity in these cases. Relative transcript abundances were determined for viruses with sufficient data. Furthermore, a multi-time-point real-time RT-PCR (RT^2^-PCR) analysis was applied to monitor the expression kinetics of the three most important lncRNAs in PRV.

We also applied RT^2^-PCR for the validation of lncRNAs and longer mRNA isoforms of PRV, BoHV-1, EHV-1, HSV-1, and KSHV. Native RNA sequencing was employed to validate the results of cDNA sequencing. CAGE-Seq was applied for the validation of KSHV and EHV-1 TSSs obtained by ONT sequencing. It is worth noting that the size-biasing effect of library preparation and LRS techniques may lead to non-detection or considerable underestimation of the abundance of long (> 5 kb) transcripts. Our RT^2^-PCR analysis demonstrated that despite this bias, the long transcripts are indeed expressed at lower levels.

### raRNAs: transcripts proximal to replication origins

Transcripts that overlap or map proximal to the Oris include lncRNAs, as well as long 5′ and 3′ UTR isoforms of mRNAs (Supplementary Table 2 and FigShare: https://doi.org/10.6084/m9.figshare.22339879.v1).

#### Alphaherpesviruses—OriS

**Figure 2:**
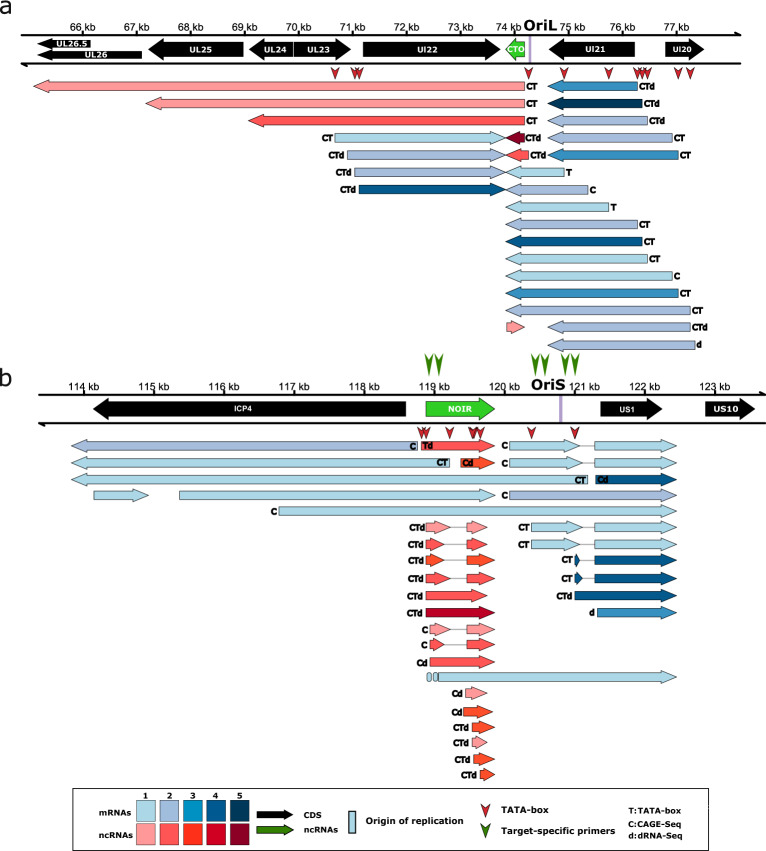
Ori-proximal transcripts of EHV-1. This figure illustrates the transcripts encoded by the OriL- (**a**) and OriS- (**b**) proximal regions of Equid alphaherpesvirus 1. For the library preparation, both polyA-selected and ribodepleted samples were used. However, in both cases, the RNAs were reverse transcribed to cDNA using an oligo(dT) primer. All the putative transcripts were identified by LoRTIA software using dcDNA datasets unless otherwise stated. Protein-coding genes are marked with black arrows, non-coding genes with green arrows, mRNAs with blue arrows, and ncRNAs with red arrows. For better comparability, we use the names for the genes applied in HSV-1 terminology. The relative transcript abundance is indicated by shading. Shades represents relative abundance: 1: 1–9 reads, 2: 10–49 reads, 3: 50–199 reads, 4: 200–999 reads, 5: > 1000 reads. CAGE-Seq was performed (transcripts detected by this technique are marked with a ‘C’). Transcripts with a proximal TATA box are labeled by a ‘T’ letter, and the vertical red arrows () show TATA box positions on the genome. Transcripts also detected by dRNA-Seq are marked with a ‘d’ letter at upstream positions. Introns are represented by horizontal lines. The position of PCR primers are indicated by vertical green arrows ().

**Figure 3:**
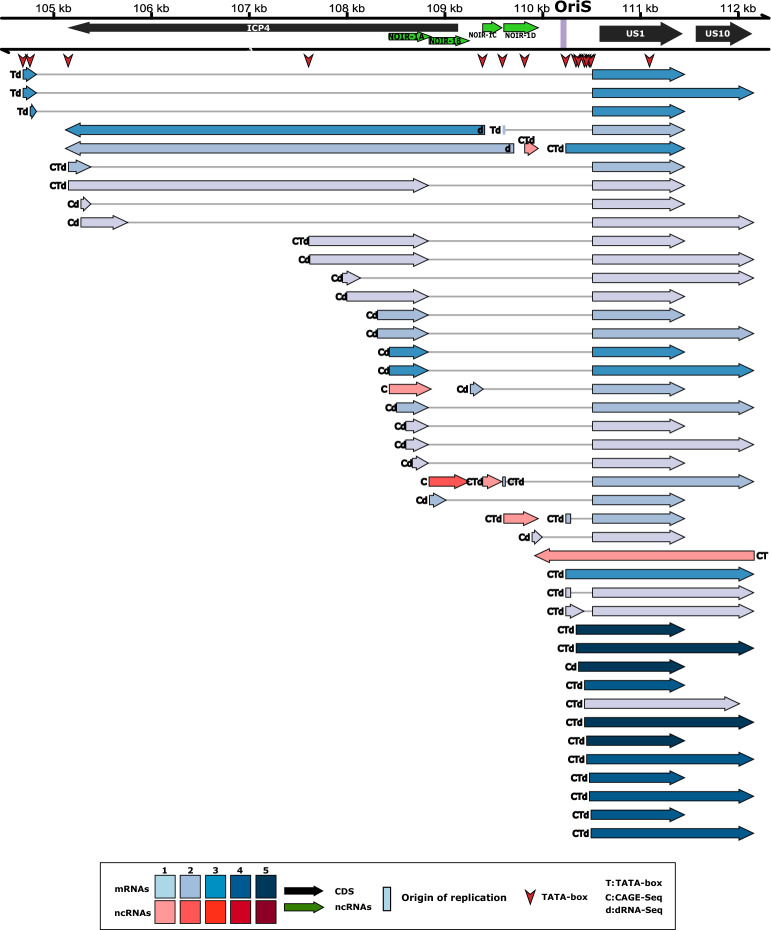
OriL-proximal transcripts of VZV. This figure shows the transcripts encoded by the OriS-proximal region of Varicella-zoster virus. All putative transcripts were identified by LoRTIA software using dcDNA datasets unless otherwise stated. Protein-coding genes are labeled with black arrows, non-coding genes with green arrows, mRNAs with blue arrows, and ncRNAs with red arrows. For better comparability, we have adopted the naming convention used for HSV-1 genes. Relative transcript abundance is indicated by shading. Shades represent relative abundance: 1: 1–9 reads, 2: 10–49 reads, 3: 50–199 reads, 4: 200–999 reads, 5: > 1000 reads. Transcripts with a proximal TATA box are marked by a ‘T’ letter, and those that were also detected by dRNA-Seq are marked with a ‘d’ letter at the upstream positions. Vertical red arrows () show the positions of TATA boxes on the genome. Transcripts detected by CAGE-Seq are marked with a ‘C’ letter. Introns are represented by horizontal lines.

In this study, we detected novel transcripts and TIs near the OriSs of α-HVs, including lncRNAs such as OriS-RNA of BoHV-1, OriS-RNA1 of HSV-1, NOIR-1 transcripts of PRV, EHV-1, VZV and Simian Varicellovirus (SVV), and NOIR-2 transcript of PRV (Figs. 2 and 3 and Supplementary Figs. 2–5). We found that in all examined αHVs, the very long 5′ TIs of transcription regulator genes (us1 and icp4) of BoHV-1, EHV-1, HSV-1, and SVV overlap the OriS. We cannot exclude the possibility that this is the case for other αHVs, but these putative transcripts might have gone undetected due to their low abundance. We detected a very complex splicing pattern of US1 transcripts in αHVs. Novel lncRNAs oriented antisense to the HSV-1 OriS-RNA1 were also identified. The NOIR-1 family members display a divergent configuration in relation to the *icp4* gene. The canonical versions of these RNAs do not overlap *icp4,* whereas the longer NOIR-1 variants partially overlap this major TR gene. In EHV-1, VZV, SVV, and probably in PRV, a long TI of US1 originates from the promoter of the *noir-1* gene. In SVV, we discovered a long TSS variant of NOIR-1 that overlaps the canonical ICP4 transcript. Both this variant and the canonical NOIR-1 transcript overlap the OriS. NOIR-1 is expressed at a moderate level, whereas NOIR-2 exhibits very low-abundance. In the same genomic region of VZV, we detected five lncRNAs with distinct TSSs and TESs designated them as NOIR-1A, -1B, 1C, -1D, and -1E. We found TIs with TSSs mapping very close to the TATA boxes within the OriSs in all six αHVs, suggesting that these promoter elements are functional.

#### Alphaherpesviruses—OriL

The CTO transcript family has been described in PRV in our earlier works^16,62^^.^ In these initial publications, we identified three 3′-coterminal transcripts: the short CTO-S, the CTO-M [initiated near the poly(A) signal of *ul21* gene], and CTO-L [a transcriptional read-through isoform (3′ UTR variant) encoded by the *ul21* gene]. It is worth noting that the long 3′ UTR isoforms with unique TES are exceedingly uncommon in αHV mRNAs. The transcript family list was later updated^55^, with a more comprehensive version provided in this current report (Supplementary Fig. 3). We also detected CTO transcripts in EHV-1 (Fig. 3), but not in other herpesviruses with annotated transcriptomes. Both PRV and EHV-1 exhibit a notably high CTO-S expression. We observed a tail-to-tail (convergent) transcriptional overlap (TO) between the 3′ UTR isoforms of CTO-S and UL22 transcripts, and identified very long read-through CTO transcripts in both viruses. We examined two PRV strains, the laboratory strain Kaplan (PRV-Ka^63^) and a field isolate (strain MdBio: PRV-MdBio^64^). In HSV-1, the divergent *ul29*-*ul30* gene pair members produce long 5′ UTR variants that overlap the OriL, but no lncRNA was found near the HSV-1 OriL.

#### Betaherpesviruses

Our data show that RNA4.9^42^ is initiated from the OriLyt of HCMV (Supplementary Fig. 6). UL59^65^, SRT^45^ and vRNA-2^46^ were also identified using our previously published dataset^36^. Additionally, we detected two longer versions of UL58 lncRNA and a shorter isoform of UL59 lncRNA.

#### Gammaherpesviruses

**Figure 4:**
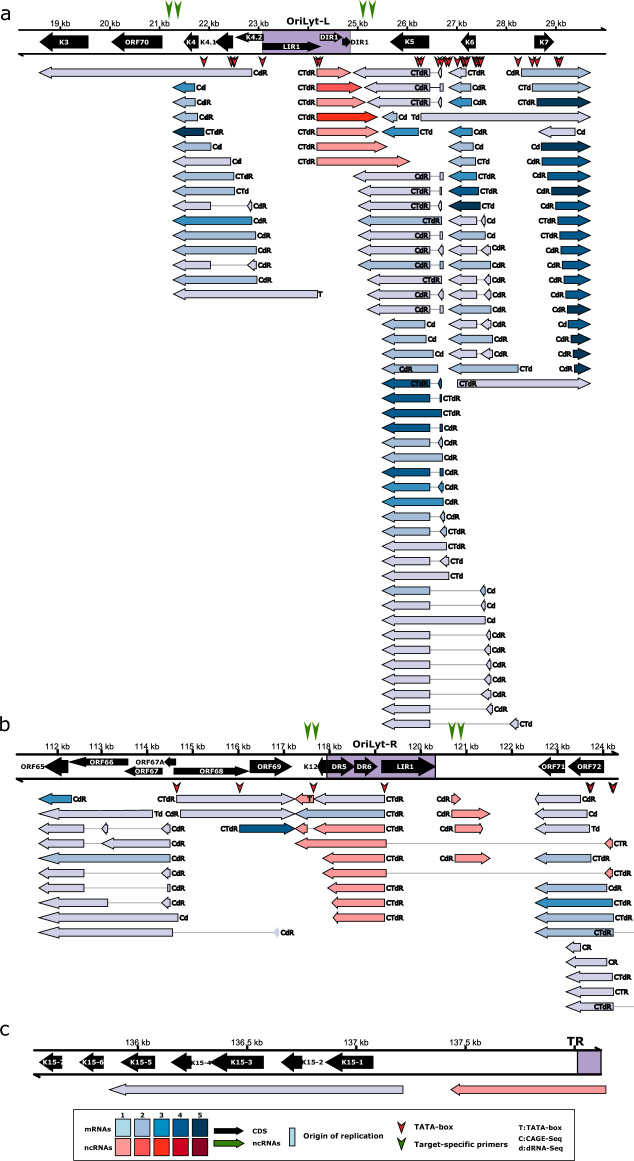
Ori-proximal transcripts of KSHV. This image illustrates the transcripts specified by the Ori-proximal regions of Kaposi’s sarcoma-associated herpesvirus (Orilyt-L: **a**; OriLyt-R: **b**; TR: **c**). All putative transcripts were identified by LoRTIA software using dcDNA datasets unless otherwise stated. Protein-coding genes are marked with black arrows, non-coding genes with green arrows, mRNAs with blue arrows, and ncRNAs with red arrows. Relative transcript abundance is indicated by shading. Shades represent relative abundance: 1: 1–9 reads, 2: 10–49 reads, 3: 50–199 reads, 4: 200–999 reads, 5: > 1000 reads. Transcripts with a proximal TATA box are marked by a ‘T’ letter, and those that were also detected by dRNA-Seq are marked with a ‘d’ letter at the upstream positions. The vertical red arrows () indicate the positions of TATA boxes on the genome. Transcripts detected by CAGE-Seq are marked by a ‘C’ letter. RAMPAGE data were also available for KSHV (transcripts detected by this technique are marked by an ‘R’ letter). Introns are represented by horizontal lines. The position of PCR primers are indicated by vertical green arrows ().

The long 5′ UTR isoform of EBV *BCRF1* gene overlaps the OriP^66^ (Supplementary Fig. 7a). Likewise, the long 5′ UTR variants of *BHRF1* gene overlap OriLyt-L. One of these transcripts is also a splice isoform encoded by this gene. The promoter of *BHLF1* gene is located within the OriLyt-L^47^. Here we describe novel isoforms of lncRNAs that either overlap the OriLyt-R with their introns or are initiated from the Ori. Our analyses revealed that several OriLyt-L-associated ncRNAs of varying length can be produced from the same TSS besides the 1.4-kb lncRNA during lytic reactivation of KSHV (Fig. 4**).** The OriLyt-L is flanked by short protein-coding genes such as K4.2, K4.1 and K4 on the left and K5, K6, and K7 on the right side^67^. Previous studies indicated that K4, K4.1, and K4.2 are transcribed as mono-, bi-, and tricistronic mRNAs^68^, but our analysis show a more complex expression pattern that includes unspliced RNAs of varying length and spliced RNA variants as well. We also found that K5/K6 genes are expressed not only individually but also through splicing, which results in mRNAs whose first exon is of different length. Importantly, our results are in line with previous transcriptomic studies^69–71^ but also expand the number of different viral RNA transcripts produced from the OriLyt-L locus. The latency locus of KSHV encode four protein-coding genes [K12, K13, ORF72, ORF73 (LANA)] and twelve pre-micro RNAs (miRNAs)^71–73^. Here, we detected several lncRNAs that are antisense to the miRNA-coding genomic regions.

### RNA isoforms of transcription regulator genes

The *us1* gene produces very long 5′ UTR isoforms, which were observed to create head-to-head TOs with ICP4 transcripts in EHV-1 (Fig. 2), VZV (Fig. 3), and HSV-1 (Supplementary Fig. 5). In HSV-1, the 5′ UTR isoforms of US10-12 polycistronic transcripts were found to establish a divergent TO with the *icp4* gene. Additionally, in HSV-1, we detected an ICP4 TIs with an extended 5′ UTR that overlaps the *us1* gene. In BoHV-1, a 3′ UTR isoform of *icp4* has been reported to create a parallel TO with the downstream *icp0* gene 1, but, the *icp4* ORF is spliced out from this transcript, resulting in a chimeric RNA containing the full-length *icp0* gene and a portion of 5′ UTR of the ICP4 transcript^74^. Additionally, ICP4 TIs were found to generate similar chimeric and bicistronic transcripts with the BoHV-1 CIRC RNA, which maps to the adjacent genomic locus in the circular or concatemeric viral genome.

### Non-coding RNAs mapping near the transcription regulator genes

In this work, we detected antisense RNAs (asRNAs) overlapping the *us1* gene in BoHV-1 and PRV. Previously, ELIE was only identified in PRV, but we found a transcript with a similar genomic location in EHV-1 (Fig. 2). ELIE is situated between the *icp4* and *icp0* genes, with one of its TIs being 5′-coterminal with NOIR-1 transcripts. We also identified an asRNA in EHV-1, named as64, which shares the same orientation as PRV ELIE but it is located within the *icp4* gene. Here, we also describe an HSV-1 transcript that starts at the 3′ end of *icp4* gene and terminated at the *us1* gene. Moreover, we detected a TSS of long 5′ UTR isoform of the VZV *us1* gene, downstream of *icp4* gene, at a position homologous to the TSS of PRV ELIE. In PRV, another lncRNA called AZURE is oriented opposite to the *us1* gene.

### Transcriptional overlaps of replication genes

In Simplexviruses, the long 5′ UTR isoforms of divergent *ul29*-*ul30* gene pair not only overlap the OriL but also each other (Supplementary Fig. 5). Intriguingly, both genes encode the main regulators of DNA replication. In both HCMV (*ul57*) and human herpesvirus type 6 (HHV-6) (*ul42*), the *ul29* orthologs are found adjacent to the OriLyt. Remarkably, in αHVs, three ‘hard’ TOs between gene pairs exist, with one partner always being a gene involved in viral replication. These TOs include *ul30*/*ul31*, *ul6*-*7*/*ul8-9*, *ul50*/*ul51* (*ul30*: DNP; *ul8*: DNA helicase; *ul9*: OBP; *ul50*: deoxyuridine triphosphatase).

### Defining the TSS patterns of examined genomic regions

**Figure 5:**
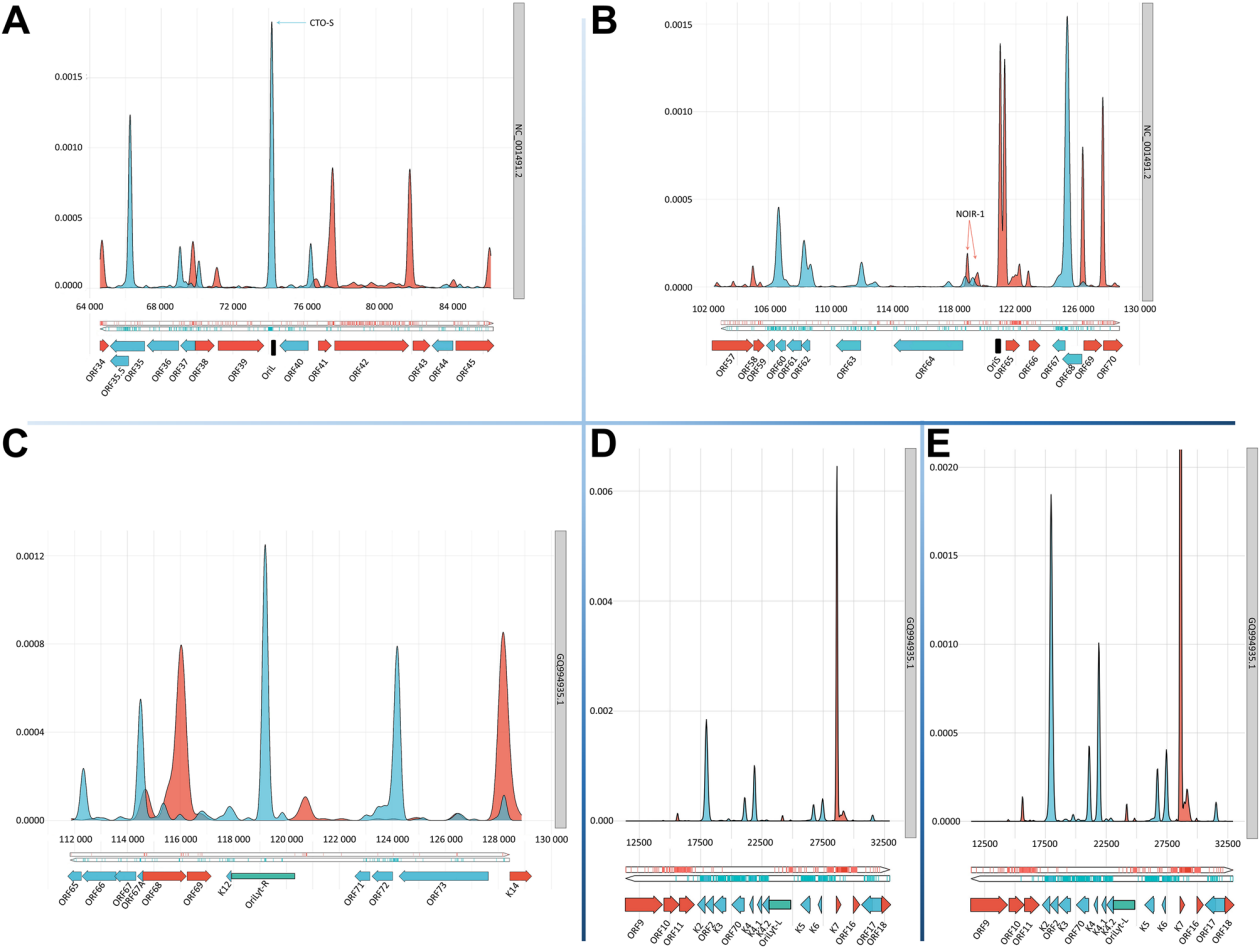
TSS Distribution in examined genomic regions determined by CAGE-Seq. TSS distributions are illustrated in the following genomic regions of EHV-1 and KSHV: (**A**) EHV-1 OriL; (**B**) EHV-1 OriS of the IRS; (**C**) KSHV OriLyt-R; (**D**) KSHV OriLyt-L; (**E**) KSHV OriLyt-L. A higher resolution is used for the better visibility of the low-abundance TSSs. CTO-S transcript is highly expressed (**A**), whereas NOIR-1 is a group of relative low-expressed transcripts (**B**). Smoothed density plots of the 5′ ends in the CAGE data. The y-axis shows the probability estimation of the 5′ ends using a probability density function (details in the Materials and Methods section). Coding sequence annotations for the respective genomes (displayed with the accession number on the right) are visualized in the lower part. Positive strand coverage and the coding sequence annotation are shown in red, while in KSHV they are depicted in blue in the negative strand. The Ori regions in EHV-1 are shown in black, whereas in KSHV they are depicted in green. In the latter case, an accompanying white box displays the 20-nt binding site for the DNA replication origin-binding protein.

Determining the TSS of RNA molecules is a significant challenge in transcriptome research. In addition to using various LRS and SRS approaches, we conducted CAGE-Seq for EHV-1 and KSHV (Fig. 5) and utilized CAGE data from others for VZV^75^ and EBV^60^ for the analysis (Figs. 2, 3 and 4). We compared our KSHV TSS CAGE-Seq results with RAMPAGE-Seq results published by others^76^. We described a total of 199 KSHV transcripts, of which 192 were confirmed by CAGE and 159 by RAMPAGE. All TSSs detected by RAMPAGE were also identified by CAGE.

### Transcript validation using qRT-PCR

**Figure 6:**
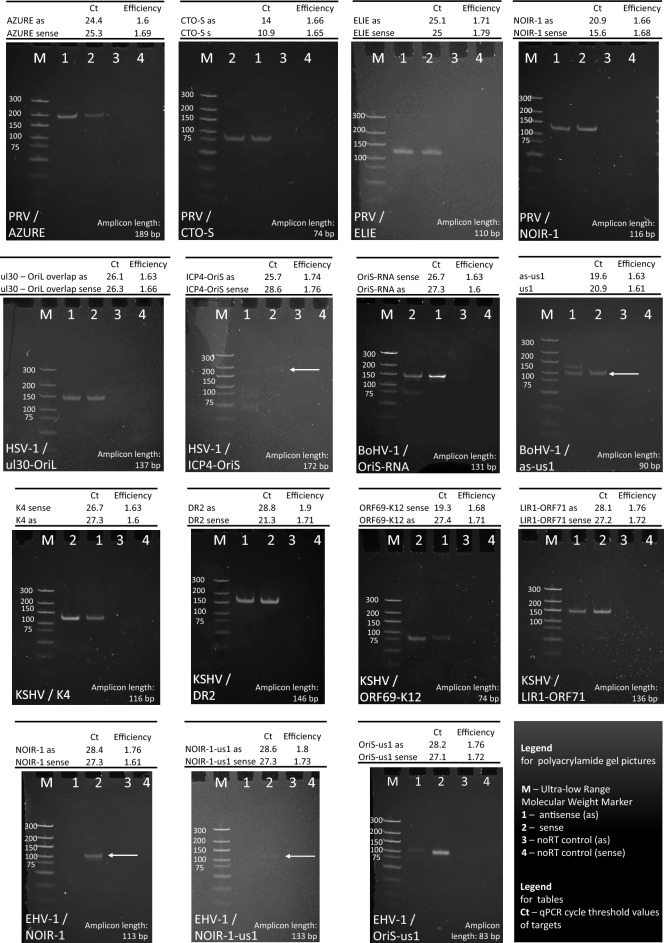
Validation of Ori-adjacent transcripts using qRT-PCR. This figure presents the validation of key Ori-adjacent transcripts using qRT-PCR and gel electrophoresis. The virus and the transcript names are indicated at the appropriate panels. We examined the transcriptional activity of both DNA strands, and used no-RT controls for each transcript. The lanes in every panel are as follows. M: molecular weight marker; 1: antisense transcripts; 2: sense transcripts (mRNA, or the canonical lncRNA); 3: no-RT for the antisense transcripts; 4. no-RT for the sense transcripts. We also indicated the amplicon lengths the Ct and efficiency values, which allow transcript quantity estimation.

Fifteen transcripts of five viruses were further validated using qRT-PCR (Fig. 6). The Ct values clearly indicate that the long TSS isoforms of the TR genes overlapping the Ori and each other are expressed at low-levels.

### Transcription kinetics of three lncRNAs of PRV

**Figure 7:**
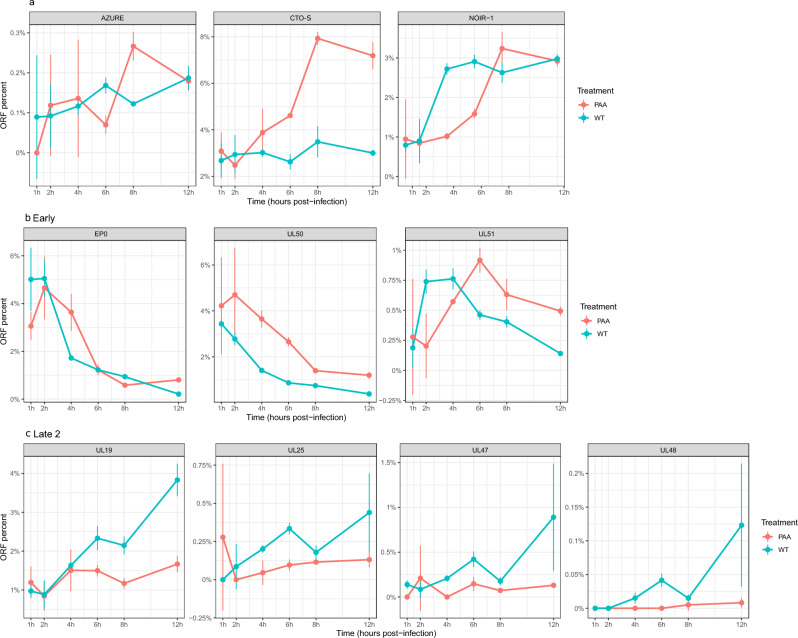
Expression kinetics of PRV transcripts in untreated and in PAA-treated samples. (**a**) Three lncRNAs: AZURE, CTO-S, NOIR-1. (**b**) Three early transcripts: EP0, UL30, UL50, UL51. (**c**) Four late (L2) transcripts: UL19, UL25, UL47, UL48. The ratios of every time points were calculated by normalizing the read count of a specific transcript against the read counts of total viral transcripts in both the untreated and PAA-treated samples. The influence of PAA-treatment was most pronounced on the L2 genes, as their expression is contingent upon DNA replication, a process inhibited by PAA. CTO-S clearly displayed early expression kinetics, whereas other two lncRNAs demonstrated complex expression dynamics.

In this work, we performed temporal sequence analyses on three PRV lncRNAs namely CTO-S, NOIR-1, and AZURE, along with three early transcripts (EP0, UL50, UL51) and four late-2 (L2) transcripts (UL19, UL25, UL47, UL48) utilizing both untreated (UT) and phosphoamino acid (PAA)-treated specimens (PAA acts as a blocker of DNA replication) (Fig. 7**, **Supplementary Table 3). Each specimen was generated in three biological replicates and assessed using dcDNA-Seq. We found that the PAA treatment suppressed the overall transcript levels; therefore, we adjusted the number of transcripts relative to the total viral RNAs. The effect of the treatment on the transcript abundancies was evaluated using the PAA/UT ratio. Logically, a value less than 1 indicates an L2 expression kinetic. Additionally, we evaluated these specimens at 12 h post-infection using qRT-PCR, yielding results that aligned closely with our findings using dcDNA-Seq analysis (Table 2). Based on our observations, it is evident that PAA treatment exerted the most pronounced effect on L2 genes, manifesting in a notable reduction in their relative abundance within the overall viral reads when compared to untreated specimens. Conversely, early transcripts manifested elevated ratios, especially during later stages, in the PAA-treated samples. While CTO-S exhibited an obvious early kinetic characteristic, the remaining two lncRNAs displayed complex expression trends. Notably, AZURE is characterized by low expression levels, which might explain its irregular expression dynamics.

**Table 2:** qRT-PCR and dcDNA-Seq analysis of PRV transcripts in PAA-treated and untreated samples.

Sample				Ct value	Average Ct value	∆Ct	∆∆Ct	Expression fold change
28S rRNA	Untreated		1	13	12.53	0.87		
			2	12.4				
			3	12.2				
	PAA		1	11.7	11.67			
			2	11.7				
			3	11.6				
ncRNAs	AZURE	Untreated	1	25.1	24.97	− 0.63	− 1.50	2.83
			2	25				
			3	24.8				
		PAA	1	25.4	25.6			
			2	26.2				
			3	25.2				
	CTO-S	Untreated	1	12.5	12.67	− 2.47	− 3.33	10.08
			2	12.9				
			3	12.6				
		PAA	1	15.4	15.13			
			2	15.2				
			3	14.8				
	NOIR-1	Untreated	1	18.3	17.77	− 3.00	− 3.87	14.59
			2	18.2				
			3	16.8				
		PAA	1	21.1	20.77			
			2	20.6				
			3	20.6				
Early genes	ep0	Untreated	1	19.2	18.97	− 0.97	− 1.83	3.56
			2	19.3				
			3	18.4				
		PAA	1	19.8	19.93			
			2	20.2				
			3	19.8				
	ul50	Untreated	1	20.7	20.53	− 0.53	− 1.40	2.64
			2	20.8				
			3	20.1				
		PAA	1	21.2	21.07			
			2	21.2				
			3	20.8				
	ul51	Untreated	1	25,3	25,3	− 0.73	− 1.60	3.03
			2	25,3				
			3	25,3				
		PAA	1	25.9	26.03			
			2	26.1				
			3	26.1				
Late-2 genes	ul19	Untreated	1	15.3	14.97	− 2.97	− 3.83	14.25
			2	15.1				
			3	14.5				
		PAA	1	18	17.93			
			2	18.2				
			3	17.6				
	ul25	Untreated	1	17.3	17.03	− 2.63	− 3.50	11.31
			2	17.3				
			3	16.5				
		PAA	1	19.8	19.67			
			2	19.9				
			3	19.3				
	ul47	Untreated	1	21.8	21.67	− 3.57	− 4.43	21.61
			2	22.2				
			3	21				
		PAA	1	25.3	25.23			
			2	25.2				
			3	25.2				
	ul48	Untreated	1	16	15.87	− 4.67	− 5.53	46.31
			2	15.9				
			3	15.7				
		PAA	1	20.6	20.53			
			2	20.7				
			3	20.3				

## Discussion

The advent of LRS technologies has made it possible to identify and precisely annotate transcripts and RNA isoforms, including length and splice variants. Technologies such as Single Molecule, Real-Time (SMRT; PacBio) sequencing^77,78^ , nanopore sequencing (ONT)^17^, and LoopSeq synthetic LRS^53^ (operating on Illumina platform; Loop Genomics) have been employed alone, or in conjunction with one other or with SRS to annotate viral transcripts in all three subfamilies of herpesviruses (HSV-1^79,80^; VZV^75,81^; PRV^16^; BoHV-1^82^; HCMV^83^; and EBV^77,84^).

In recent studies, we and others have demonstrated the existence of a previously hidden, highly complex, genome-wide network of transcriptional overlaps in different viral families^23,75,77,80,82^. It has been shown that the RNA molecules encoded by closely spaced genes overlap each other in a parallel, divergent, or convergent manner. This phenomenon implies an interaction between the transcription machineries at the TOs throughout the entire viral genome^85^. We and others have previously demonstrated that in several viruses, the replication origins overlap with specific lncRNAs and with long 5′ or 3′ UTR isoforms of mRNAs^86^. We would like to point out that numerous 5′ UTR isoforms might be non-coding, as there is a considerable distance between their TSSs and the start codons. Exceptions may be those transcripts whose large parts of the 5′ UTR are spliced out. Functional analyses have revealed the mechanistic details of how replication RNAs control the onset of DNA synthesis through the formation of RNA:DNA hybrids in several viruses^43^.

In this work, we present a state-of-the-art annotation of Ori-adjacent transcripts that potentially play key roles in regulating the replication and/or genome-wide transcription of herpesviruses. We put more emphasis on the examination of αHVs because less information is available for such transcripts in these viruses. Using novel and previously published sequencing data, we discovered new transcripts and corrected the earlier annotations of already described RNAs. Furthermore, we identified an intricate meshwork of TOs between transcripts encoded by replication and transcription regulatory genes and also the specific lncRNAs around them. Promoter consensus elements within the Oris were also detected in all examined herpesviruses. Although herpesvirus Oris contain AT-rich sequences, which can be misidentified as TATA boxes, we detected the corresponding TSSs being proximal to these sequences in all cases. The terminology of the reported lncRNAs is somewhat arbitrary because their sequences and exact locations are in many cases poorly conserved. Therefore, it is possible that some of these lncRNAs with the same name have a polyphyletic origin. Nevertheless, the CTO and NOIR-1 transcript families are apparently orthologous in PRV and EHV-1.

**Figure 8:**
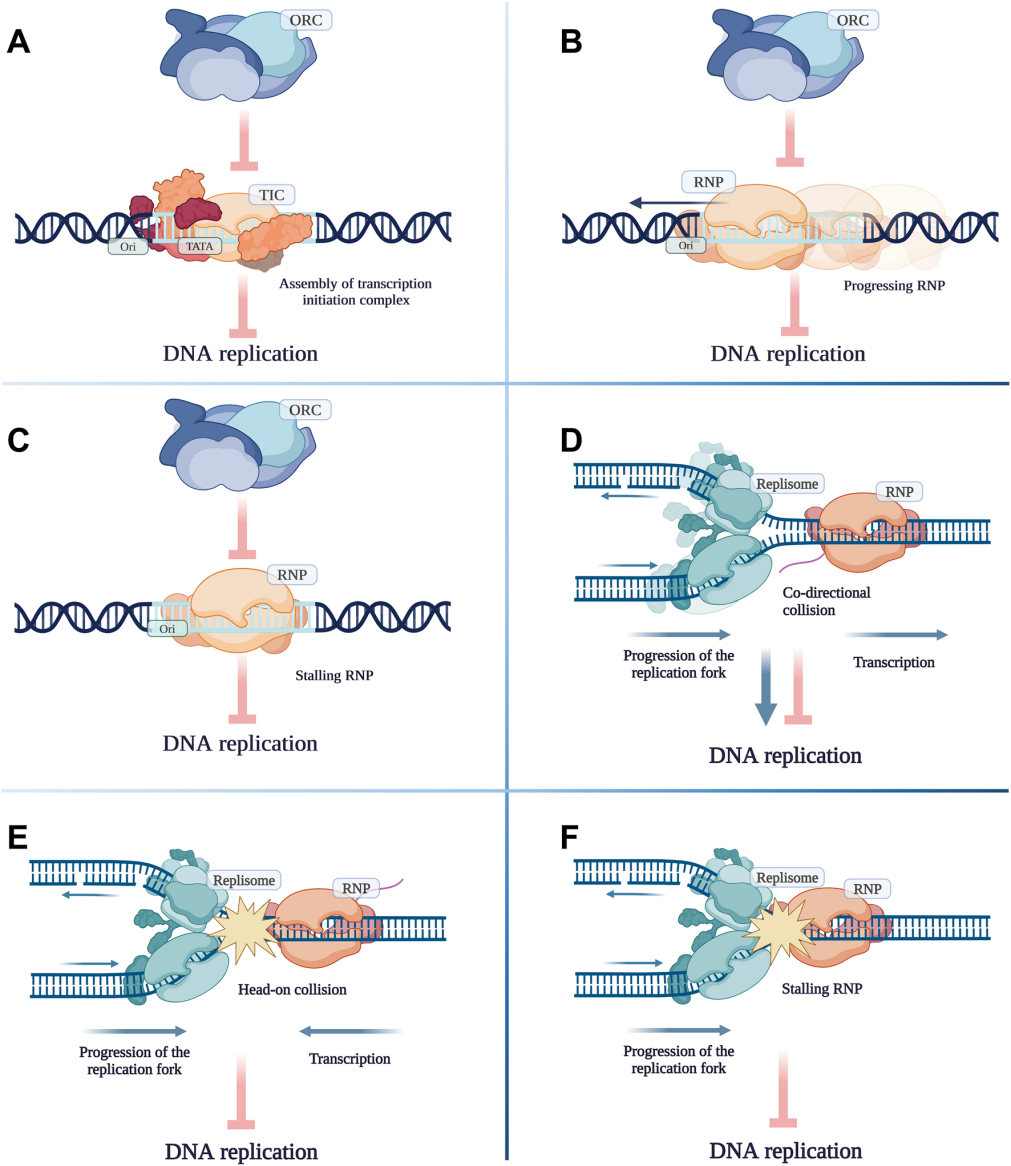
Potential effects of transcription on the DNA replication. (**A**) Recruitment of transcription initiation complex on promoters located within the replication origin inhibits assembly of ORC and replisome. (**B**) RNA polymerase II passage across the replication origin inhibits of ORC and replisome assembly. (**C**) RNA polymerase II stalling on the replication origin inhibits ORC and replisome assembly. (**D**) Co-directional movement of replication and transcription machineries can slow down or speed up replication fork progression. Transcription can facilitate DNA replication by pre-opening the two DNA strands. (**E**) Head-on collision of replication and transcription machineries inhibits both processes. (**F**) RNA polymerase II stalling inhibits replication fork progression.

The co-temporal activity of DNA replication and transcription within the same genomic region generates interference between the two processes along the entire genome^87^. Evidence suggests that the collisions have more dramatic consequences when they occur in convergent orientation rather than co-directionally^88^. Several molecular mechanisms have evolved to minimize the conflict between the RNP and the replication fork^89^. However, our current study suggests the existence of an in-built, intrinsic regulatory system based on an interaction between the two apparatuses for controlling the initiation of both replication and transcription cooperatively. This interaction is thought to be mediated through the clash and competition between the transcribing RNP and the replisome, as well as the assembly of pre-replication and transcription initiation complexes by the ongoing DNA and RNA syntheses (Fig. 8 and Supplementary Figs. 8 and 9).

In αHVs, the position of OriS is highly conserved within the genome: it is located in the US repeats and surrounded by the major TF genes, *icp4* and *us1*. Here, we demonstrate that TF genes produce transcript isoforms that overlap not only the OriS but also the TF transcripts of at the other side of the replication origin, and/or specific proximal lncRNAs. The probable function of the TOs is to facilitate additional forms of interplay between these genes (asides from the TF/promoter interaction), which may occur through RNA:DNA and possibly by RNA:RNA hybridization, as well as interference between the transcription apparatuses. As a result, the US-IR region of αHVs appears to function as a ‘super regulatory center’, where the initiation of both DNA replication and global transcription is collectively controlled via a mutually interacting multilevel system. In addition, this genomic segment governs the transition between the lytic cycle and latency, as well as the maintenance of these processes. Hence, this region is functionally the most complex genomic locus of αHVs, encoding lncRNAs such as intergenic transcripts and asRNAs, long TIs of mRNAs, and an intricate TO pattern of local transcripts. Besides the lytic transcripts, several latent lncRNAs (LAT, LLT, L/ST) and miRNAs are also expressed from this genomic region^90^.

The NOIR-1 transcript family, mapped to the US-IR region, is the evolutionary innovation of the Varicellovirus genus. The precise location of these lncRNA molecules exhibits significant variation. In PRV, the long TIs of NOIR-1 overlap the *icp4*, but no OriS overlapping NOIR-1 TES isoforms have been detected in this virus. A possible explanation for this is that in PRV this transcript is opposed to NOIR-2 whose expression might prevent transcriptional read-through by NOIR-1 toward the OriS. The PRV NOIR-1 is 3′ co-terminal with the LLT transcripts expressed during latency. In EHV-1, the NOIR-1 long TI overlaps with *icp4*, while the long TSS isoform of US1 transcript, driven by the NOIR-1 promoter, overlaps the OriS. In VZV and SVV, the NOIR-1 promoters also control the transcription of the long TSS variants of US1. Additionally, in VZV, the upstream NOIR-1 transcripts also overlap the *icp4*. Intriguingly, in SVV, the NOIR-1 terminates at the OriS, which appears to be a unique solution for the interplay between the two machineries.

BoHV-1 lacks NOIR-1, but has a long TSS isoform of US1 transcripts that overlaps the OriS. Additionally, BoHV-1 expresses OriS-RNA, which has an orientation parallel to *icp4*. In HSV-1, the OriS-RNA1 has an opposite polarity to NOIR-1 and is situated on the other side of the OriS. The promoter of this transcript also controls the long TSS isoform of the ICP4 transcript. NOIR-1 transcripts may be involved in controlling transcription through overlapping the *icp4* gene, and/or in controlling DNA replication through overlapping the OriS (in cases when they are the upstream part of the US1 TI), or in controlling both transcription and replication (in cases when they overlap both OriS and *icp4*). The same might also be true for the HSV-1 OriS-RNA1, as it also serves as the 5′ UTR part of the long ICP4 TI.

It may not be a coincidence that the OriL of Simplexviruses is surrounded by the main replication genes (*ul29/30*). We hypothesize that these genes might interact with initiation process of replication not only through conventional TF/promoter binding but also at other levels, including the RNA:DNA and/or RNA:RNA hybridization, and through interference between transcription and replication machineries at the overlapping region. The CTO transcripts have been previously identified in PRV^51^. Here, we report the detection of the orthologous transcripts in EHV-1. The canonical CTO-S does not overlap the OriL, but it might interfere with replication by helping to separate the two DNA strands and thereby determining the orientation of replication, or in other ways. One of the long CTO-S TIs is controlled by a promoter within the OriL, and another one is the TES variant of *ul21* gene.

We also observed a potentially intriguing phenomenon: in αHVs, a total of three ‘hard’ convergent TOs are formed, which may represent a strong confrontation between transcription apparatuses during the synthesis of RNA molecules. We noticed that one of the partners forming these TOs is always an auxiliary replication gene. The involvement of such genes in every ‘hard’ TOs may not be just a mere coincidence but have an unknown biological relevance. We can speculate that the ‘hard’ TO between the *ul30****/****ul31* partner genes of Simplexviruses allows *ul31* gene to generate an OriL-overlapping long TES isoform through transcriptional read-through. Indeed, we detected a high-frequency transcriptional read-through from this gene using RT^2^-PCR in PRV. Interestingly, although in PRV and EHV-1 the OriL is located between the *ul21* and *ul22* genes, the OriL is also overlapped by a long TES isoform of *ul21*. Since no long TES isoforms of *ul21* in Simplexviruses and of *ul31* in Varicelloviruses are produced, it is reasonable to assume that the role of these types of TIs is to interfere with the initiation of DNA replication.

The HCMV OriLyt is overlapped by lncRNAs, among which RNA4.9 has been shown to regulate DNA replication^43^. We have described novel Ori-overlapping lncRNAs of HCMV previously^36^ and in this recent report. The OriLyt-L of EBV is adjacent to the non-coding BHLF1 gene, a latency regulator^91^, while the OriLyt-R is adjacent to LF2, an inhibitor of replication^92^. KSHV LANA plays an essential role as a latency regulator^93^. In this study, we describe additional Ori-overlapping transcripts, including lncRNAs and long 5′ UTR isoforms of mono- and polycistronic mRNAs.

We quantified the amount of particularly long 5′UTR variants using qRT-PCR and found that their low abundance cannot be explained solely by the size-bias of the sequencing; these transcripts are indeed expressed at a low level. Although many of them carry complete ORFs, they are probably untranslated since their transcription and translation start sites are far apart. Thus, they might be transcriptional by-products without functioning as protein-coding sequences, or even as RNA molecules.

In summary, even closely related herpesvirus species have developed unique strategies for establishing transcriptional overlaps (TOs) at the Oris and the TR genes. This underscores the significance of this phenomenon in regulating DNA replication and overall transcription. While the major TR genes appear to control each other and the initiation of DNA replication at the OriS region of αHVs, the main replication genes at the OriL region of Simplexviruses, might rather regulate each other and DNA replication through mechanisms achieved via TOs. These putative mechanisms provide multiple regulatory layers in addition to conventional transcription factor/promoter interaction. ICP4 has been shown to promote the expression of *icp0*^94^ genes by binding to its promoter. ICP22 (*us1* product) inhibits the expression of both *icp4* and *icp0*^95^. Furthermore, ICP0 converts ICP4 from a repressor to an activator of mRNA synthesis in HSV-1^96^. The mutation of *us1* gene leads to a differential effect on the transcription kinetics of E and L genes^97^.

We believe that the implications of our results have a much broader perspective and represent a general way of how the regulation of herpesviral replication and transcription co-evolved with each other. Understanding these complex interactions between various genes and regulatory elements may provide valuable insights into the overall regulatory mechanisms governing herpesvirus replication and gene expression. Further investigation into these potential interactions and their functional significance may open the door for the creation of innovative therapeutic approaches to address herpesvirus infections.

## Methods

### Cells and viruses

In addition to our novel data, we also used several other datasets for the analyses in this study. The cell types used in this work are listed in Supplementary Table 4.

***PRV*** For the generation of novel transcript data, we employed three immortalized cell lines to propagate the MdBio strain of pseudorabies virus (PRV-MdBio^64^): PK-15 porcine kidney epithelial cell line (ATCC® CCL-33™), C6 cell line derived from a rat glial tumor (ATCC® CCL-107™), and PC-12 (ATCC® CRL-1721™) derived from a pheochromocytoma of the rat adrenal medulla, which have an embryonic origin from the neural crest. Each experiment was conducted in three biological replicate. PK-15 cells were cultured in Dulbecco’s modified Eagle medium (DMEM) (Gibco/Thermo Fisher Scientific), supplemented with 5% fetal bovine serum (FBS; Gibco/Thermo Fisher Scientific) and 80 μg of gentamycin per ml (Gibco/Thermo Fisher Scientific) at 37 °C in the presence of 5% CO_2_. C6 cells were cultivated in F-12 K medium (ATCC), complemented with 2.5% FBS and 15% horse serum (HS; Sigma-Aldrich) at 37 °C in the presence of 5% CO_2_. PC-12 cells were maintained in RPMI-1640 medium (ATCC) supplemented with 5% FBS and 10% HS at 37 °C in the presence of 5% CO_2_. Virus stock solution was prepared as follows: PK-15 cells were infected with 0.1 multiplicity of infection [MOI = plaque-forming units (pfu)/cell]. Viral infection was allowed to progress until complete cytopathic effect was observed, which was followed by three successive cycles of freezing and thawing of infected cells in order to release of viruses from the cells. Each cell type was infected with MOI = 1 of PRV-MdBio. Infected cells were incubated for 1 h at 37 °C followed by removal of the virus suspension and washing the cells with phosphate-buffered saline (PBS). Following the addition of new culture medium, the cells were incubated for 0, 1, 2, 4, 6, 8, or 12 h. Following the incubation, the culture medium was discarded and the infected cells were frozen at − 80 °C until further use.

For the kinetic analysis, we used MOI = 1 of PRV-Ka for the infection of PK-15 cells. Cells were first incubated at 4 °C for 1 h for synchronization of infection, and then placed in a 5% CO_2_ incubator at 37 °C. Infected cells were collected at every 30 min within an 0–8 h interval. Cells were washed with PBS, scraped from the culture plate, and centrifuged at 3000 RPM for 5 min at 4 °C.

***EHV-1*** Equid alphaherpesvirus 1 was also employed in this study. A field isolate of the virus (EHV-1-MdBio) was used, which is originated from Marócpuszta (Hungary) from the organs of an aborted colt fetus in the 1980s. A confluent rabbit kidney (RK-13) epithelial cell line (ECACC 0,021,715) was used for viral propagation. The experiments were carried out in three technical replicates.

RK-13 cells were maintained in DMEM (Sigma). The media was supplemented with 10% fecal calf serum (FCS, Gibco). The culture conditions were as follows: 37 °C in the presence of 5% CO_2_. A virus stock solution was prepared by infecting the cells with EHV-1-MdBio at MOI = 0.1. Three freeze–thaw cycles were applied to release the viruses from the cells. For the EHV-1 long-read RNA-seq investigations, RK-13 cells were infected with MOI = 4 of the virus. Three technical replicates were carried out. Viral infected RK-15 cells were incubated at 4 °C for 1 h, then the virus suspension was removed and cells were washed with PBS. Next, new media was added to the cells, which were incubated for 1, 2, 4, 6, 8, 12, 18, 24 or 48 h. Finally, the culture medium was removed from the cells and they were stored at − 80 °C until further use.

***KSHV***: The KSHV-positive primary effusion lymphoma cell line iBCBL1-3xFLAG-RTA^98^ was maintained in RPMI 1640 medium, which was supplemented with 10% Tet System Approved FBS (TaKaRa), penicillin/streptomycin, and 20 μg/ml hygromycin B. KSHV lytic reactivation was induced by treating 1 million of iBCBL1-3xFLAG-RTA cells with 1 μg/ml doxycycline for 24 h. For measuring KSHV gene expression by qRT-PCR, cDNA was generated with iScript cDNA Synthesis kit (Bio-Rad) followed by SYBR green-based real-time quantitative PCR analysis using gene specific primers. The relative viral gene expression was calculated by the delta-delta Ct method where 18S was used for normalization. The sequences of the primers have been reported previously (Toth et al., 2013). The following antibodies were used for immunoblots: anti-FLAG (Sigma F1804), anti-ORF6 (from Dr. Gary S. Hayward, Johns Hopkins University), and anti-Tubulin (Sigma T5326).

***VZV***: Human primary embryonic lung fibroblast cell line (MRC-5) obtained from the American Type Culture Collection (ATCC) was used for the propagation of the live attenuated OKA/Merck strain of varicella zoster virus. Cells were grown in DMEM supplemented with antibiotic/antimycotic solution and 10% fetal bovine serum (FBS) at 37 °C in a 5% CO_2_ atmosphere. Infected cells were harvested by trypsinization when they displayed cytopathic effect (after 5 days).

**HSV-1:** An immortalized kidney epithelial cell line (Vero) was used for the propagation of HSV-1. The cell culture was grown in DMEM (Gibco/Thermo Fisher Scientific) with 10% Fetal Bovine Serum (Gibco/Thermo Fisher Scientific) and 100 μl/ml penicillin–streptomycin Mixture (Lonza), and in a 37 °C incubator with a humidified atmosphere of 5% CO_2_ in air. Cells were infected with HSV-1 at an MOI = 1, then they were incubated for 1 h. The virus suspension was removed and cells were washed with PBS. This step was followed by the addition of fresh medium to the cells and they were incubated for 1, 2, 4, 6, 8, or 12 h. A mixture from the different time points were prepared for the further analysis.

**BoHV-1:** Madin–Darby Bovine Kidney (MDBK) cells were used for the infection using the Cooper isolate (GenBank Accession # JX898220.1) of Bovine alphaherpesvirus 1.1. Cells were incubated at 37 °C in a humidified incubator with 5% CO_2_ and were cultured in DMEM supplemented with 5% (v/v) fetal bovine serum, 100 U/mL penicillin, and 100 µg/mL streptomycin. Cells were infected using MOI = 1 virus suspension. Infection was allowed to progress until complete cytopathic effect was observed. Infected cells were incubated for 1, 2, 4, 6, 8, and 12 h, then the supernatant was collected, and the cellular fraction was subjected to three successive cycles of freezing and thawing in order to release additional intracellular virions.

### Inhibition of DNA synthesis using phosphonoacetic acid

Before the infection, we treated the cells with 400 µg/ml PAA (phosphonoacetic acid) for 1 h at 37 °C in the presence of 5% CO_2_ to inhibit replication and determine the kinetic class of transcripts. During the experiment, we examined the cells at six different time points (1, 2, 4, 6, 8, 12 h) using three biological replicates. For the treated samples, we also used an MOI of 10 of the virus for infection. RNA was isolated at 1, 2, 4, 6, 8, 12 h post-infection time points, and a dcDNA library was prepared according to the library preparation protocol of the direct cDNA Sequencing Kit (SQK-DCS109). We sequenced the samples on Oxford Nanopore MinION flow cells. Subsequently, the data were basecalled by Guppy, mapped using minimap2 software, and further analyzed using scripts deposited on GitHub.

(https://github.com/Balays/Rlyeh?fbclid=IwAR0HZJNXZjv9YUm_tsJ5J1eT2fKXnkhbJKf7WVoTxX9kvp7fJmdhWQILbjA). Data have been deposited under the project accession number PRJEB64684 into the European Nucleotide Archive (ENA).

### RNA isolation

***Extraction of total RNA*** Total RNA was isolated from the PRV, EHV-1, VZV and KSHV infected cells by using the NucleoSpin® RNA kit (Macherey–Nagel). The spin-column protocol was applied. In brief, cells were lysed by the addition of a chaotropic ion containing buffer solution (from the kit). Nucleic acids were then bound to a silica membrane. Samples were treated with DNase I to remove genomic DNA. Total RNAs were eluted with RNase-free water. To eliminate the potential remaining DNA from the samples, we used the TURBO DNA-free™ Kit. Samples were stored at -80 °C.

***Purification of polyadenylated RNA*** The Qiagen Oligotex mRNA Mini Kit was used to enrich the mRNAs (and other RNAs with polyA-tail) from the PRV, EHV-1 and VZV samples, which were then used as templates for ONT and Illumina library preparations. The Spin Columns protocol of the manual was applied. In brief, the final volume of the RNA samples was set to 250 µL by adding RNase-free water. Then, 15 µL Oligotex suspension and 250 µL OBB buffer (both from the Oligotex kit) were added to the mixtures, which were first incubated at 70 °C for 3 min and then at 25 °C for 10 min. Samples were centrifuged at 14,000 × g for 2 min, and the supernatants were removed. Four-hundred µL Oligotex OW2 wash buffer was added to the samples, then they were spun down in Oligotex spin columns at 14,000 × g for 1 min. This step was repeated once, and finally, the poly(A) + RNA fraction was eluted from the membrane by adding 60 µl hot Oligotex elution buffer. To maximize the yield, a second elution step was also carried out. The Lexogen Poly(A) RNA Selection Kit V1.5 was used for the selection of polyadenylated RNAs from the KSHV total RNA samples. Briefly, 10 µL of total RNA (5 µg) was denatured at 60 °C for 1 min then it was hold at 25 °C. The RNA samples were mixed with 10 µL washed bead (including in the kit) and the mixtures were incubated at 25 °C for 20 min with 1,250 rpm agitation. Afterwards, the tubes were placed in a magnetic rack for 5 min. Supernatant was discarded and the beads were resuspended in Bead Wash Buffer (supplied by the Lexogen kit) and were incubated 25 °C for 5 min with 1250 rpm agitation. Tubes were transferred onto the magnetic rack and supernatant was discarded after 5 min incubation. This washing step was carried out twice. After the second washing step, the beads were resuspended in Nuclease-free water (Lexogen kit) and then incubated at 70 °C for 1 min. Then, the tubes were transferred onto the magnet for 5 min. The supernatant containing the poly(A) + RNA fraction was transferred into a fresh tube.

***Removal of rRNA*** Ribo-Zero Magnetic Kit H/M/R (Epicentre/Illumina) was used to remove ribosomal RNAs and to enrich mRNAs. Unlike poly(A) + purification, the rRNA depletion process also retains RNAs without polyA tails, except for rRNAs. For starting material, 5 µg of a mixture of EHV-1 total RNA was used. The sample was mixed with the Ribo-Zero Reaction Buffer and Ribo-Zero rRNA Removal Solution. The mixture was incubated at 68 °C for 10 min, then at room temperature for 5 min. Next, 225 µl washed Magnetic Bead was added to the sample and they were incubated at room temperature for 5 min, then at 50 °C for 5 min. Finally, the mixture was placed on a magnetic stand, then the supernatant containing the rRNA-depleted RNA was collected. The final purification was carried out by using the Agencourt RNAClean XP Beads (Beckman Coulter) as recommended by the Ribo-Zero manual.

***Enrichment of the 5*′ *ends of RNAs*** Terminator™ 5′-Phosphate-Dependent Exonuclease (Lucigen) was used to enrich the 5′ ends of the transcripts. The process was carried out with a mixture of poly(A) + RNAs from the EHV-1 samples, which was mixed with Terminator 10X Reaction Buffer A, RiboGuard RNase Inhibitor and Terminator Exonuclease (1 Unit). The mixture was incubated at 30 °C for 60 min, then the reaction was stopped by the addition of 1 µL of 100 mM EDTA (pH 8.0). RNAClean XP beads (Beckman Coulter) was used for final purification.

### Measurement of nucleic acid quality and quantity

***RNA:*** The Qubit 4. fluorometer and the Qubit Assay Kits (Invitrogen) were used for the measurement of RNA concentration. The RNA BR Assay was applied for the quantitation of total RNA samples whereas the RNA HS Assay was utilized for the poly(A) + and ribodepleted RNA fractions. The quality of the total RNA samples was checked by using the Agilent TapeStation 4150 device, RNA ScreenTape and reagents were used. RIN scores above 9.6 were used for cDNA production. The RNA quality was assessed with the Agilent 2100 Bioanalyzer (for PacBio sequencing) or Agilent 4150 TapeStation System (for MinION sequencing) and RIN scores above 9.6 were used for cDNA production.

***cDNA:*** The Qubit dsDNA HS Assay Kit (Invitrogen) was used to quantify the cDNA samples. For the analysis of Illumina library quality, the Agilent High Sensitivity D1000 ScreenTape was used.

### Direct cDNA sequencing

Libraries were created without PCR amplification from the poly(A) + fractions of RNAs from PRV, EHV-1 and KSHV as well as from the Terminator-treated EHV-1 samples. To achieve this, the ONT’s Direct cDNA Sequencing Kit (SQK-DCS109) was utilized according to the ONT’s manual. Briefly, the RNAs were combined with ONT VN primer and 10 mM dNTPs, and incubated at 65 °C for 5 min. Subsequently, RNaseOUT (Thermo Fisher Scientific), 5 × RT Buffer (Thermo Fisher Scientific), ONT Strand-Switching Primer were added to the mixtures, which were then incubated at 42 °C for 2 min. Maxima H Minus Reverse Transcriptase enzyme (Thermo Fisher Scientific) was added to the samples to produce the first cDNA strands. The reaction was conducted at 42 °C for 90 min, and the reactions were halted by heating the samples at 85 °C for 5 min. The RNAs from the RNA:cDNA hybrids were removed using RNase Cocktail Enzyme Mix (Thermo Fisher Scientific). This reaction took place at 37 °C for 10 min. The LongAmp Taq Master Mix [New England Biolabs (NEB)] and ONT PR2 Primer were used to synthetize the second strand of cDNAs. The following PCR condition was applied: 1 min at 94 °C, 1 min 50 °C, then 15 min at 65 °C. As a next step, the end-repair and dA-tailing were carried out with the NEBNext End repair /dA-tailing Module (NEB) reagents. These reactions were conducted at 20 °C for 5 min, followed by heating the samples at 65 °C for 5 min. Adapter ligation was then carried out with the NEB Blunt /TA Ligase Master Mix (NEB) at room temperature for 10 min. The ONT Native Barcoding (12) Kit was utilized to label the libraries, then they were loaded to ONT R9.4.1 SpotON Flow Cells (200 fmol mixture of libraries was loaded to one flow cell. AMPure XP Beads were used after each enzymatic step, and samples were eluted in UltraPure™ nuclease-free water (Invitrogen).

### Amplified Nanopore cDNA sequencing

For more accurate mapping of the 5′-end of VZV transcripts, random-primer based RT reactions were carried out as a first step of library preparation. For this, poly(A)-selected and Terminator-treated RNA samples and SuperScript IV enzyme (Life Technologies) were used. From the first-strand cDNAs, libraries were prepared according to the modified 1D strand switching cDNA by ligation protocol Ligation Sequencing kit (SQK-LSK108; Oxford Nanopore Technologies). KAPA HiFi DNA Polymerase (Kapa Biosystems) and Ligation Sequencing Kit Primer Mix (part of the 1D Kit) were used to amplify the cDNAs. Next, samples were end-repaired using NEBNext End repair /dA-tailing Module (New England Biolabs), then adapter ligation was carried out using the sequencing adapters supplied with the kit and NEB Blunt/TA Ligase Master Mix (New England Biolabs).

### Short-read sequencing

For the SRS transcriptomic analysis of EHV-1 virus, libraries were generated from a mixture of poly(A) + enriched and rRNA-depleted samples using the NEXTflex® Rapid Directional qRNA-Seq Kit (PerkinElmer). Fragmentation of the RNAs was carried out enzymatically at 95 °C for 10 min via the addition of NEXTflex® RNA Fragmentation Buffer. Next, the first strand cDNA was synthetized. First, the NEXTflex® First Strand Synthesis Primer was mixed with the RNA sample, which were incubated at 65 °C for 5 min, then the mixture was subsequently placed on ice. RT reaction was performed by using the NEXTflex® Directional First Strand Synthesis Buffer and Rapid Reverse Transcriptase according the following protocol: incubation at 25 °C for 10 min, then at 50 °C for 50 min, and termination at 72 °C for 15 min. Generation of the second cDNA strand was carried out at 16 °C for 60 min with the addition of NEXTflex® Directional Second Strand Synthesis Mix (with dUTPs). Adenylation of cDNAs were performed at 37 °C for 30 min using the NEXTflex® Adenylation Mix. The reaction was terminated by incubating the samples at 70 °C for 5 min. As a next step, Molecular Index Adapters (part of the Kit) were ligated to the sample using the NEXTflex® Ligation Mix. Ligation was carried out at 30 °C for 10 min. Samples were amplified by PCR. As a first step of this, the NEXTflex® Uracil DNA Glycosylase was added to the samples, and then they were incubated at 37 °C for 30 min, which was followed by heating at 98 °C for 2 min, and they were subsequently placed on ice. Then, the following components were added to the samples: PCR Master Mix, qRNA-Seq Universal forward primer, and qRNA-Seq Barcoded Primer (sequence: AACGCCAT; all from the PerkinElmer kit). The following settings were applied for the PCR: 2 min at 98 °C, followed by 15 cycles of 98 °C for 30 s, 65 °C for 30 s and 72 °C for 60 s, followed by a final extension at 72 °C for 4 min. AMPure XP Bead was used after each enzymatic reaction. The NEXTflex® Resuspension buffer was used for the final elution of the sequencing library from which 10 pM was loaded to the Illumina MiSeq reagent cassette. Paired-end transcriptome sequencing was performed on an Illumina MiSeq sequencer by using the MiSeq Reagent Kit v2 (300 cycles).

### Direct RNA sequencing

Native RNA sequencing approach was also applied for library preparation from the EHV-1and KSHV samples with the aim to avoid potential biases associated with reverse transcription (RT) and PCR reactions to detect and validate novel splice variants as well as 3′ UTR isoforms. For the dRNA-seq experiments, we used an RNA mixture containing RNA from each time points of infection from the Poly(A) + samples and from the Poly(A) + and Terminator-treated RNAs. The oligo dT-containing T10 adapter for RT priming and the RNA CS for monitoring the sequencing quality (both from the ONT kit) were added to the RNA mix along with NEBNext Quick Ligation Reaction Buffer, and T4 DNA ligase (both from NEB). The reaction was incubated for 10 min at room temperature. Then, 5 × first-strand buffer, DTT (both from Invitrogen), dNTPs (NEB) and UltraPure™ DNase/RNase-Free water (Invitrogen) were added to the samples. Finally, SuperScript III enzyme (Thermo Fisher Scientific) was mixed with the sample and RT reaction was performed at 50 °C for 50 min, and subsequently stopped by heat inactivation of the enzyme at 70 °C for 10 min. RNA adapter (from the ONT kit) was ligated to the RNA:cDNA hybrid sample using the NEBNext Quick Ligation Reaction Buffer and T4 DNA ligase at room temperature for 10 min. The RNAClean XP Beads were used after each additional enzymatic step. Two flow cells were used for dRNA-seq, 100 fmol from the sample was loaded onto each of them.

### Real-time RT-PCR

Quantitative qRT-PCR was used for transcript validation and kinetic analysis, and was carried out as previously described^99^ using the primers listed in Supplementary Table 5. Briefly, first strand cDNAs from the total RNAs of PRV, EHV-1, VZV, HSV-1, BoHV-1 and KSHV were synthesized by using the SuperScript III reverse transcriptase and gene-specific primers (ordered from Integrated DNA Technologies). The reactions were carried out in 5 μl of final volume containing 0.1 μg of total RNA, 2 pmol of the primer, dNTP mix (Invitrogen, 10 μM final concentration), 5 × First-Strand Buffer, and the RT enzyme (Invitrogen). The reaction was performed at 55 °C for 60 min and then terminated by heating the samples to 70 °C for 15 min. Ten-fold dilutions from the RT products were used as template for real-time PCR amplification. PCR reactions were carried out using ABsolute QPCR SYBR Green Mix (Thermo Fisher Scientific) in a Rotor-Gene Q (Qiagen) cycler. The running conditions are shown in Supplementary Table 6. The following controls were used: loading control (28S rRNA as a reference gene), no template (checking the potential primer-dimer formation) and no RT control (to detect the potential DNA contamination). The relative expression levels (R) were calculated using the mathematical framework proposed by Soong and colleagues^100^. However, we made some adjustments: the R values were computed using the mean ECt value from the 6 h samples for every gene as a benchmark. This was then normalized against the mean value of the corresponding 28S values.

We used the Comparative Quantitation module of the Rotor-Gene Q software package that automatically calculates the qRT-PCR efficiency sample-by-sample and set the cycling thresholds values automatically.

$${\text{R}} = \begin{array}{*{20}c} {\overline{{\left( {E_{{sample6h}} } \right)^{{Ct_{{sample6h}} }} }} } \\ {\overline{{\left( {E_{{sample}} } \right)^{{Ct_{{sample}} }} }} } \\ \end{array} :\begin{array}{*{20}c} {\overline{{\left( {E_{{sample6h}} } \right)^{{Ct_{{ref6h}} }} }} } \\ {\overline{{\left( {E_{{sample6h}} } \right)^{{Ct_{{ref}} }} }} } \\ \end{array}$$

We used the 2^-ΔΔCt^ method for the comparison of gene expression values of the PAA-treated samples with the untreated samples^101^.

#### Cap Analysis of Gene Expression

We employed CAGE-Seq to investigate the TSS distribution patterns at the examined genomic regions in EHV-1 and KSHV using three biological replicates. The CAGE™ Preparation Kit (DNAFORM, Japan) was utilized. CAGE libraries were prepared from 5 µg of total RNA following the manufacturer’s recommendations. Briefly, the RNA and the RT primer (random primer mixture from CAGE™ Prep Kit) was denatured at 65 °C for 5 min. First cDNA strands were synthesized by using SuperScript III Reverse Transcriptase (Invitrogen). To enhance the activity and specificity of RT enzyme, trehalose/sorbitol mixture (CAGE™ Prep Kit) was also added. The reactions were incubated for 30 s at 25 °C, then the RT reaction was performed at 50 °C for 60 min. Diol groups in the Cap at the 5′-end (and ribose at 3′-end) were oxidized with NaIO_4_ and Biotin (long arm) hydrazine was bound to it. First, the oxidation was carried out with the addition of NaOAc (1 M, pH 4.5, CAGE™ Prep Kit) and NaIO_4_ (250 mM, CAGE™ Prep Kit) to the samples and incubation on ice for 45 min in the dark. After this step, 40% glycerol and Tris–HCl (1 M, pH 8.5, CAGE™ Prep Kit) were added to the samples. NaOAc (1 M, pH 6.0) and Biotin Hydrazine (10 mM, CAGE™ Prep Kit) were mixed with the samples and the oxidized diol residues were biotinylated at 23 °C for 2 h. After this step, single strand RNA was digested by applying RNase I (CAGE™ Prep Kit) treatment (37 °C 30 min). Biotinylated, Capped RNA samples were mixed and bound (Cap-trapping) to the pretreated Streptavidin beads (pretreatment details below) at 37 °C 30 min. Then they were incubated on a magnetic rack. The beads were washed with Wash Buffer 1 (twice), then with Wash Buffer 2, and finally with Wash Buffer 3 (both from the CAGE™ Prep Kit). Next, cDNAs were released from the beads: Releasing Buffer was added to the samples and they were incubated at 95 °C for 5 min. After a short incubation on a magnetic rack, the supernatant (containing the Capped cDNAs) was transferred to new tubes. RNase I buffer (CAGE™ Prep Kit) was added to the tRNA-Streptavidin bead and they were placed on a magnetic rack. The supernatant was transferred to the tubes containing the cDNAs and they were stored on ice. The samples were treated with an RNase mixture (RNase H and RNase I, both from the CAGE™ Prep Kit) and incubated at 37 °C for 15 min. The potential remaining RNA was digested with RNase I. The reaction was performed at 37 °C for 30 min.

Streptavidin beads were coated with tRNA (CAGE™ Prep Kit), followed by mixing and incubation on ice for 30 min, and then incubated on a magnetic stand for 3 min. The supernatant was removed, and the beads were washed with Wash Buffer 1 (CAGE™ Prep Kit) twice. Finally, the beads were eluted in Wash Buffer 1 and tRNA was also added. The volume of the samples was reduced by using the miVac DUO Centrifugal Concentrator (Genevac), then single strand 5′ linkers (with barcodes) were ligated to the samples at 16 °C for 16 h using the DNA ligation mixture CAGE™ Prep Kit). After a purification step, the miVac DUO was used again in order to concentrate the samples. This was followed by the ligation of the 3′ linker using the DNA ligation mixture. It was carried out at 16 °C for 16 h. (The 5′ and 3′ linkers were pre-heated at 55 °C and the cDNA samples at 95 °C before the ligation steps). The samples were mixed with Shrimp Alkaline Phosphatase (SAP, CAGE™ Prep Kit) to remove the phosphate group of the linkers. The reaction was conducted at 37 °C for 30 min and terminated at 65 °C for 15 min. Then USER enzyme was added to the sample, which digests the dUTP from 3′ linker up strand. The USER treatment was carried out at 37 °C for 30 min and it was stopped at 95 °C for 5 min. After this step, the barcoded samples were mixed and concentrated with miVac DUO. Finally, the second cDNA strands were synthesized with the 2nd primer, DNA polymerase, buffer and 10 mM dNTP (all from the CAGE™ Prep Kit). The denaturation step was set to 95 °C for 5 min, the annealing to 55 °C for 5 min, and the elongation to 72 °C for 30 min. The sample mixture was treated with Exonuclease I enzyme (37 °C for 30 min). The vacuum concentrator was used to completely dry the sample and finally it was dispensed in 10 µl of nuclease-free H_2_O. The amount of single-stranded cDNAs was measured using Qubit 2.0 and the Qubit ssDNA HS Assay Kit. The RNAClean XP Beads were used after RT, oxidation and biotinylation. AmpureXP Beads were used to purify the samples after Cap-trapping and Releasing, RNase I treatment, 5′ and 3′ linker ligation, SAP and USER treatments, 2nd strand cDNA synthesis and Exonuclease I treatment. Libraries with different barcodes were pooled and applied on the same flow cells. The libraries were sequenced on a MiSeq instrument with v3 (150 cycles) and v2 (300 cycles) chemistries (Illumina). Qubit 4.0 and 1X dsDNA High Sensitivity (HS) Assay was used to measure the concentration of the sample. The quality of the library was tested by TapeStation.

## Bioinformatics analyses

### Illumina CAGE sequencing data analysis

Read quality was checked with **fastqc** (https://www.bioinformatics.babraham.ac.uk/projects/fastqc). The reads were trimmed with **TrimGalore** (https://github.com/FelixKrueger/TrimGalore) using the following settings: *-length 151 -q*. The **STAR** aligner^102^, version 2.7.3.a was used to map the reads to the KSHV strain TREx reference genome (*GQ994935.1*) using *–genomeSAindexNbases 8,* otherwise default parameters. The **CAGEfightR** R package^103^ was used to identify TSSs and TSS clusters using a minimum pooled value cutoff of 0.1 (*pooledcutoff* = *0.1*).

### ONT sequencing data analysis

**Guppy** software (v3.4.5) was used to basecall the ONT-MinION sequencing reads. Reads passing the quality filter of 8 (default), were mapped to the reference genome using **minimap2**^104^ with the following settings: -*ax splice -Y -C5 -cs.* **ReadStatistics** script from **Seqtools** (https://github.com/moldovannorbert/seqtools) was used to compute mapping statistics. The **LoRTIA** toolkit (alpha version, accessed on 20 August 2019, https://github.com/zsolt-balazs/LoRTIA) was used to identify TESs, TSSs and introns and to subsequently reconstruct transcripts using these features.

The LoRTIA workflow with default settings was as follows: 1.) for dRNA and dcDNA sequencing: − *5 TGCCATTAGGCCGGG*—*five_score 16*—*check_in_soft 15−3 AAAAAAAAAAAAAAA –three_score 16 s Poisson–f true*; and 2.) for o(dT)-primed cDNA reads: − *5 GCTGATATTGCTGGG – five_score 16 –check_in_soft 15 − 3 AAAAAAAAAAAAAAA –three_score 16 s Poisson–f true.*

In case of EHV-1, the searching for adapter sequences was performed by the following command: *samprocessor.py*—*five_adapter GCTGATATTGCTGGG*—*five_score 14*—*check_in_soft 15*—*three_adapter AAAAAAAAAAAAAAAAAAA –three_score 14 input output*. The next step in the workflow was the annotation of TSS and TES. For the TES positions, the wobble has been increased to 20, the TSS wobble value remains the default. Then following parameters were used on the ‘sam’ files: *Stats.py -r genome -f r5 -b 10 and Stats.py -r genome -f l5 -b 10* for the TSS detection; while *Stats.py -r genome-f r3-b 20* and *Stats.py-r genome-f l3-b 20* for TES detection; and *Stats.py -r genome -f in* for intron detection.

Several sequencing techniques were used in the analysis of BoHV-1, the LoRTIA workflow was set up differently depending on sequencing techniques. The following parameters were used for dRNA sequencing: *LoRTIA -5 AGAGTACATGGG –five_score 16 –check_in_soft 15 -3 AAAAAAAAAAAAAAAAAAAAA –three_score 12 -s poisson -f True*. For oligo d(T) cDNA sequencing: *LoRTIA -5 TGCCATTAGGCCGGGGG –five_score 14*—*check_in_soft 15–3 AAAAAAAAAAAAAAAAAAAAA*—*three_score 14-s poisson-f True.* During random primer cDNA sequencing: *LoRTIA -5 TGCCATTAGGCCGGGGG*—*five_score 14*—*check_in_soft 15 -3 GAAGATAGAGAGCGACA*—*three_score 14-s poisson -f True.* Also for dcDNA sequencing: *LoRTIA -5 GCTGATATTATTGCTGGG*—*five_score 16 –check_in_soft 15–3 AAAAAAAAAAAAAAAAAAAAAAA –three_score 14 -s poisson -f True.*

A TSS was accepted if the adapters were correct while TESs were accepted if polyA tails were present and there were no false priming events detected by LoRTIA. Further, in the case of KSHV, EBV, EHV-1, TSS were accepted only if at least one dcDNA read, and one dRNA or CAGE read validated it. In the case of CMV, BoHV-1, PRV and HSV-1, TSSs were accepted only if they were present in at least three different samples. In case of introns, we accepted only those that were present in the dRNA sequencing, as this method is considered to be the ‘*Gold Standard*’ in identifying alternative splicing events. Several transcripts were manually included if they were a long TSS variant of already accepted TSSs. **MotifFinder** from **Seqtools** was used to find promoter elements around the accepted TSSs.

### Downstream data analysis and visualization

Downstream data analysis was carried out within the R environment, using **GenomicRanges**^105^, **tidygenomics**^106^ and packages from the **tidyverse**^107^. **Gviz**^108^ was used to create Figs. 2, 3 and 4— (https://github.com/ivanek/Gviz). Figure 5 was created using a custom R workflow. Briefly, the ‘.bam’ files were imported into R using **Rsamtools** (https://bioconductor.org/packages/Rsamtools). The coverage was calculated and the 5′ ends were summed per genomic position in each sample and finally an area-plot was generated using **ggplot2's** geom_area geom function. The density along with the genome annotation was plotted using a custom plotting function, utilizing gggenes (https://github.com/wilkox/gggenes). These scripts are used to import other alignments into R and to generate similar plots from their 3′ or 5′ distribution or coverage on the reference genomes as well (GitHub: https://github.com/Balays/Rlyeh).

### Supplementary Information

Supplementary Legends.

Supplementary Figure 1.

Supplementary Figure 2.

Supplementary Figure 3.

Supplementary Figure 4.

Supplementary Figure 5.

Supplementary Figure 6.

Supplementary Figure 7.

Supplementary Figure 8.

Supplementary Figure 9.

Supplementary Table 1.

Supplementary Table 2.

Supplementary Table 3.

Supplementary Table 4.

Supplementary Table 5.

Supplementary Table 6.

## Data Availability

Our data used in this study has been deposited to in European Nucleotide Archive (ENA) under the following accessions: PRJEB24593 (https://www.ebi.ac.uk/ena/browser/view/PRJEB24593), ERP106430 (https://www.ebi.ac.uk/ena/browser/view/PRJEB24593), PRJEB33511 (https://www.ebi.ac.uk/ena/browser/view/PRJEB33511), PRJEB38992 (https://www.ebi.ac.uk/ena/browser/view/PRJEB38992), PRJEB22072 (https://www.ebi.ac.uk/ena/browser/view/PRJEB22072), PRJEB25680 (https://www.ebi.ac.uk/ena/browser/view/PRJEB25680), ERP019579 (https://www.ebi.ac.uk/ena/browser/view/PRJEB17709), PRJEB25401 (https://www.ebi.ac.uk/ena/browser/view/PRJEB25401), PRJEB25433 (https://www.ebi.ac.uk/ena/browser/view/PRJEB25433), PRJEB9526 (https://www.ebi.ac.uk/ena/browser/view/PRJEB9526), PRJEB12867 (https://www.ebi.ac.uk/ena/browser/view/PRJEB12867) and to Gene Expression Omnibus (GEO) under the accession number: GSE97785 (https://www.ncbi.nlm.nih.gov/geo/query/acc.cgi?acc=GSE97785). We also acquired datasets from other groups, which were downloaded from GEO: GSE79337 (https://www.ncbi.nlm.nih.gov/geo/query/acc.cgi?acc=GSE79337), GSE59717 (https://www.ncbi.nlm.nih.gov/geo/query/acc.cgi?acc=GSE59717), GSE128324 (https://www.ncbi.nlm.nih.gov/geo/query/acc.cgi?acc=GSE128324), from Sequence Read Archive: PRJNA505045 (https://www.ncbi.nlm.nih.gov/bioproject/PRJNA505045/), PRJNA482043 (https://www.ncbi.nlm.nih.gov/bioproject/?term=PRJNA482043), PRJNA483305 (https://www.ncbi.nlm.nih.gov/bioproject/?term=PRJNA483305), PRJNA533478 (https://www.ncbi.nlm.nih.gov/bioproject/?term=PRJNA533478), and from ENA: PRJEB27861 (https://www.ebi.ac.uk/ena/browser/view/PRJEB27861), PRJEB42868 (https://www.ebi.ac.uk/ena/browser/view/PRJEB42868), PRJEB38829 (https://www.ebi.ac.uk/ena/browser/view/PRJEB38829). https://www.ebi.ac.uk/ena/browser/view/PRJEB64684?fbclid=IwAR2U3rT3i0MTVwb2xYpi9I92DOuuyuvLA8aAtWD48Qip6tw11cxpczt_hLg, PRJEB64684.

## Abbreviations

αHV: Alphaherpesvirus
BoHV-1: Bovine alphaherpesvirus 1
dRNA-Seq: Direct RNA sequencing
EBV: Epstein-Barr virus
HCMV: Human cytomegalovirus
IE: Immediate-early
IRL: Internal repeat of UL region
LAT: Latency-associated transcript
LRS: Long-read sequencing
ncRNA: Non-coding RNA
ORF: Open reading frame
PRV: Pseudorabies virus
SRS: Short-read sequencing
TF: Transcription factor
TR: Transcription regulator
US: Unique short
asRNA: Antisense RNA
DBP: DNA-binding protein
dcDNA-Seq: Direct cDNA sequencing
EHV-1: Equid alphaherpesvirus 1
HHV-6: Human herpesvirus 6
ICP: Infected cell polypeptide
IRS: Internal repeat of US region
LLT: Long latency transcript
L/ST: L/S junction-spanning transcript
ONT: Oxford nanopore technologies
Ori: Replication origin
raRNA: Replication origin-associated RNA
SVV: Simian varicella virus
TI: Transcript isoform
TSS: Transcript start site
UTR: Untranslated region
βHV: Betaherpesvirus
DNP: DNA polymerase
E: Early
γHV: Gammaherpesvirus
HSV-1: Herpes simplex virus 1
IR: Inverted repeat
L: Late
lncRNA: Long noncoding RNA
miRNA: Micro RNA
ORC: Origin recognition complex
PacBio: Pacific biosciences
RNP: RNA polymerase
TES: Transcript end site
TO: Transcriptional overlap
UL: Unique long
VZV: Varicella-zoster virus

## Code availability

LoRTIA: https://github.com/zsolt-balazs/LoRTIA. R scripts: https://github.com/Balays/Rlyeh. R workflow: https://github.com/Balays/KSHV_RNASeq.
