# Supplementary material for: Identification of herpesvirus transcripts from genomic regions around the replication origins
Source: Sci Rep. 2023 Sep 29;13:16395. doi: 10.1038/s41598-023-43344-y (PMC10541914; doi:10.1038/s41598-023-43344-y)
Supplement: Supplementary file 1 — Supplementary Legends. [file 41598_2023_43344_MOESM1_ESM.docx]

**Additional files**

**Supplementary Figure 1. Replication origin positions on herpesvirus genomes**

This figure shows the locations of inverted repeats and replication origins in the herpesvirus genomes examined in this study. *Abbreviations* Ul: unique long, US: unique short, IRS: internal repeat of US region, TRS: terminal repeat of US region, IRL: internal repeat of UL region, TRL: terminal repeat of UL region

**Supplementary Figure 2. Ori-proximal transcripts of BoHV-1**

This image displays the transcripts encoded by the OriS-proximal region of Bovine alphaherpesvirus 1 (IRS: **a.**; TRS: **b.**). All potential transcripts were identified by LoRTIA software using dcDNA datasets unless indicated otherwise. Protein-coding genes are labeled with black arrows, non-coding genes with green arrows, mRNAs with blue arrows, and ncRNAs with red arrows. For better comparability, we use the names for the genes applied in HSV-1 terminology. Relative transcript abundance is indicated by shading. Shades represent relative abundance: 1: 1-9 reads, 2: 10-49 reads, 3: 50-199 reads, 4: 200-999 reads, 5: >1000 reads. The coverage of dRNA-Seq data in BoHV-1 is relative low therefore, undetection certain RNAs with this technique does not necessarily mean low reliability. The presence a proximal TATA box is marked with a ‘T’ letter, and those that were also detected by dRNA-Seq are marked with a ‘d’ letter at the upstream positions. Vertical red arrows indicate the positions of TATA boxes on the genome. Introns are indicated by horizontal lines.

**Supplementary Figure 3. Ori-proximal transcripts of PRV**

This image displays the transcripts encoded by the OriL- (**a**) and OriS-proximal regions (**b**) of Pseudorabies virus. All putative transcripts were identified by LoRTIA software using dcDNA datasets unless indicated otherwise. Protein-coding genes are marked with black arrows, non-coding genes with green arrows, mRNAs with blue arrows, and ncRNAs with red arrows. For better comparability, we have adopted the naming convention used for HSV-1 genes. Relative transcript abundance is indicated by shading. Shades represent relative abundance 1: 1-9 reads, 2: 10-49 reads, 3: 50-199 reads, 4: 200-999 reads, 5: >1000 reads. Transcripts with a proximal TATA box are marked by a ‘T’ letter, and the vertical red arrows indicate the positions of TATA boxes on the genome. Those transcripts that were also detected by dRNA-Seq are marked with a ‘d’ letter at the upstream positions. Introns are indicated by horizontal lines.

**Supplementary Figure 4. Ori-proximal transcripts of SVV**

This figure shows the transcripts encoded by the OriS-proximal region of Simian varicella virus. All putative transcripts were identified by LoRTIA software using dcDNA datasets unless indicated otherwise. Protein-coding genes are labeled with black arrows, non-coding genes with green arrows, mRNAs with blue arrows, and ncRNAs with red arrows. For better comparability, we employ the HSV-1 naming conventions for the genes. Relative transcript abundance is indicated by shading. Shades represent relative abundance: 1: 1-9 reads, 2: 10-49 reads, 3: 50-199 reads, 4: 200-999 reads, 5: >1000 reads. Vertical red arrows indicate the positions of TATA boxes on the genome.

**Supplementary Figure 5. Ori-proximal transcripts of HSV-1**

This figure shows the transcripts encoded by the Ori-proximal regions (OriL: **a.**; OriS of IRS: **b.**; OriS of TRS: **c.**) of Herpes simplex virus 1. All the putative transcripts were identified by LoRTIA software using dcDNA datasets unless indicated otherwise. Protein-coding genes are labeled with black arrows, non-coding genes with green arrows, mRNAs with blue arrows, and ncRNAs with red arrows. Relative transcript abundance is indicated by shading. Shades represent relative abundance: 1: 1-9 reads, 2: 10-49 reads, 3: 50-199 reads, 4: 200-999 reads, 5: >1000 reads. Transcripts with a proximal TATA box are marked by a ‘T’ letter, and vertical red arrows indicate the positions of TATA boxes on the genome. Those transcripts that were also detected by dRNA-Seq are marked with a ‘d’ letter at the upstream positions. The striped ends of certain arrows (illustration of transcripts) indicate that these terminals have been not or not accurately annotated.

**Supplementary Figure 6. Ori-proximal transcripts of HCMV**

This figure shows the transcripts specified by the Ori-proximal region of Human cytomegalovirus. All the putative transcripts were identified by LoRTIA software using dcDNA datasets unless indicated otherwise. Protein-coding genes are labeled with black arrows, non-coding genes with green arrows, mRNAs with blue arrows, and ncRNAs with red arrows. Relative transcript abundance is indicated by shading. Shades represent relative abundance: 1: 1-9 reads, 2: 10-49 reads, 3: 50-199 reads, 4: 200-999 reads, 5: >1000 reads. Vertical red arrows indicate the positions of TATA boxes on the genome.

**Supplementary Figure 7. Ori-proximal transcripts of EBV**

This figure shows the transcripts specified by the Ori-proximal regions of Epstein-Barr virus (OriP: **a.**; Orilyt-L: **b.**; OriLyt-R: **c.**). All putative transcripts were identified by LoRTIA software using dcDNA datasets unless indicated otherwise. Protein-coding genes are labeled with black arrows, non-coding genes with green arrows, mRNAs with blue arrows, and ncRNAs with red arrows. Relative transcript abundance is indicated by shading. Shades represent relative abundance: 1: 1-9 reads, 2: 10-49 reads, 3: 50-199 reads, 4: 200-999 reads, 5: >1000 reads. Vertical red arrows indicate the positions of TATA boxes on the genome. In the case of very long transcripts, the names of the genes overlapped by these transcripts are enlisted. Introns are indicated by horizontal lines. All TSS of EBV transcripts have been validated by CAGE analysis, therefore this information is not indicated at the figure.

**Supplementary Figure 8. The operation of the Putative Super Regulatory Center**

**a.** Passage of RNA polymerases across the OriS by reading the long 5′ UTR of *us1* and *icp4* genes inhibits the assembly of ORC and replisome. Stalling of RNP on OriS leads to the same consequences.

**b.** Reading the long 5′ UTRs of *us1* and *icp4* genes by RNP inhibits each other expressions which exert an effect on the global transcription. Inhibition of *icp4* expression through interference between RNPs reading the two genes leads a decreased expression in most of the herpesvirus genes, including *us1* gene. Inhibition of *us1* gene expression ICP22 inhibits *icp4* expression and leads a more complex effect on genome-wide gene expression (Takács et al., 2013).

**c.** Transcriptional overlapping of replication origin may lead to the formation of RNA:DNA hybrids and thereby interfering (inhibiting or perhaps facilitating) the assembly of the replisome.

**d.** Transcriptional overlapping of replication origin and the transcription regulatory genes may lead to the formation of RNA:DNA hybrids and thereby interfering (inhibiting or perhaps facilitating) the assembly of the replisome and also the transcription.

**Supplementary Figure 9. Interference between the replication and transcription apparatuses**

**a.** Passage of RNA polymerases across the OriS by reading the long 5′ UTR of *ul29* and *ul30* genes inhibits the assembly of ORC and replisome. Stalling of RNP on OriS leads to the same consequences.

**b.** Head-on collision between the two replication genes leads to the inhibition of each other transcription and the initiation replication because of the passage of RNPs across the OriL. The replication is also affected by the inhibition of the generation of replication proteins.

**c.** Formation of RNA:DNA hybrids at the OriL interferes with the initiation of DNA replication.

**d.** Formation of RNA:DNA hybrids at the OriL and the replication genes interferes with both the initiation of DNA replication and the transcription of replication genes. The replication is also affected by the inhibition of the generation of replication proteins.

**Supplementary Table 1.** **Correspondence of orthologous transcripts**

**Supplementary Table 2. Transcripts with putative regulatory functions**

The numbers represent the count of transcripts identified at a specific time point, treatment, etc. for a particular transcript.

**Supplementary Table 3. Expression kinetics of PRV transcripts in untreated and in PAA-treated samples**

The ratios of every time points were calculated by normalizing the read count of a specific transcript against the read counts of total viral transcripts in both the untreated (UT) and PAA-treated samples. The effect of the PAA on the transcript levels was assessed using the PAA/UT ratio. A value less than 1 indicates an L2 expression kinetic.

**Supplementary Table 4. List of the cell types used in this study**

**Supplementary Table 5. List of the primers used for qRT-PCR**

**Supplementary Table 6. Running conditions of qRT-PCR**

The annealing temperature was increased to 65°C for the amplification of PRV ELIE, HSV-1 ICP4-OriS and KSHV LIR1-ORF71 transcripts.
