# Supplementary figures and images for: Identification of herpesvirus transcripts from genomic regions around the replication origins
Source: Sci Rep. 2023 Sep 29;13:16395. doi: 10.1038/s41598-023-43344-y (PMC10541914; doi:10.1038/s41598-023-43344-y)

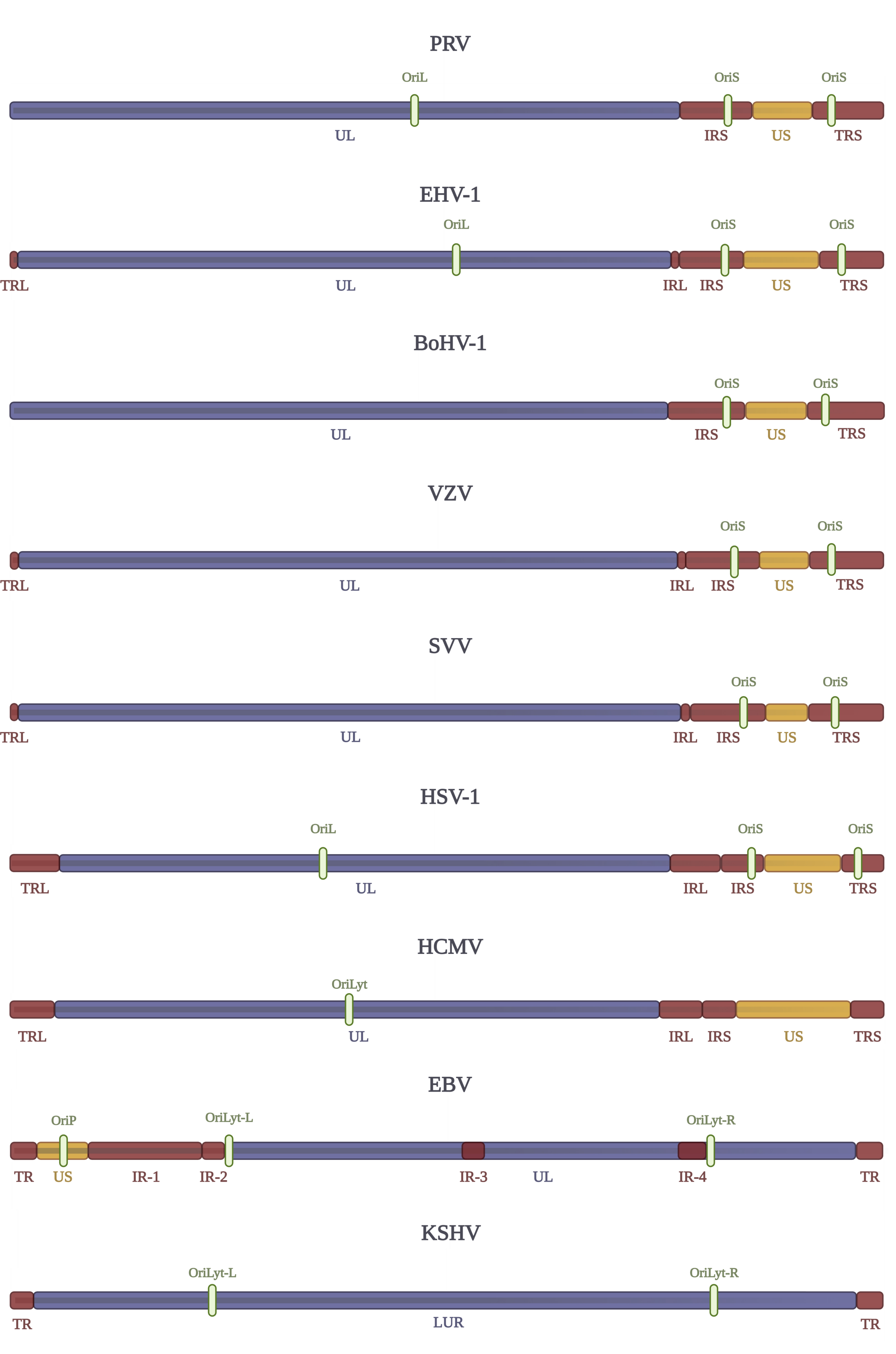

Supplement: Supplementary file 2 — Supplementary Figure 1. [file 41598_2023_43344_MOESM2_ESM.pdf]

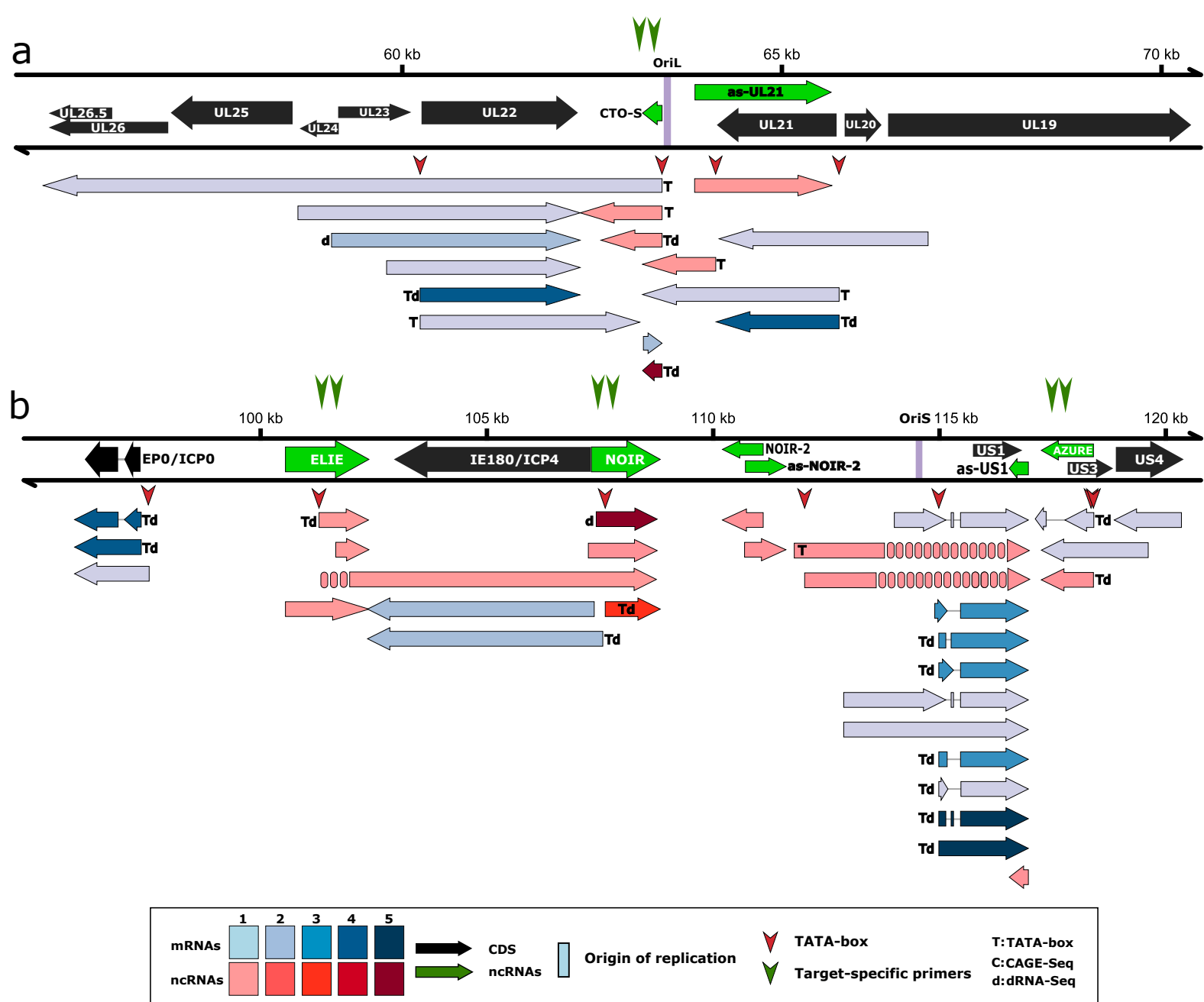

Supplement: Supplementary file 4 — Supplementary Figure 3. [file 41598_2023_43344_MOESM4_ESM.pdf]

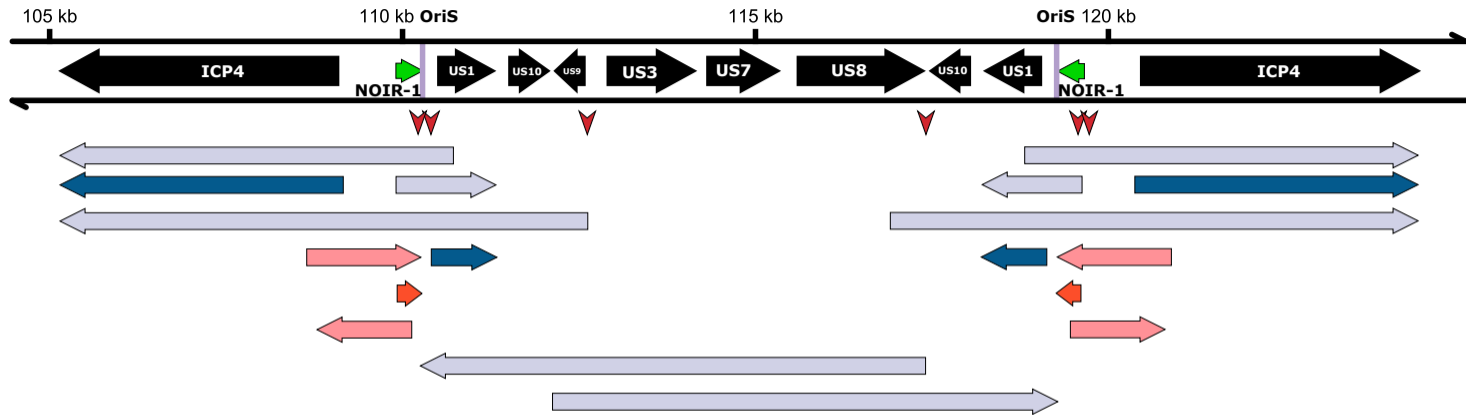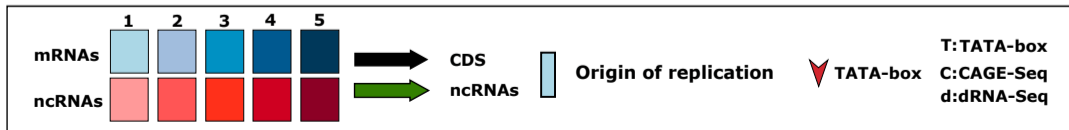

Supplement: Supplementary file 5 — Supplementary Figure 4. [file 41598_2023_43344_MOESM5_ESM.pdf]

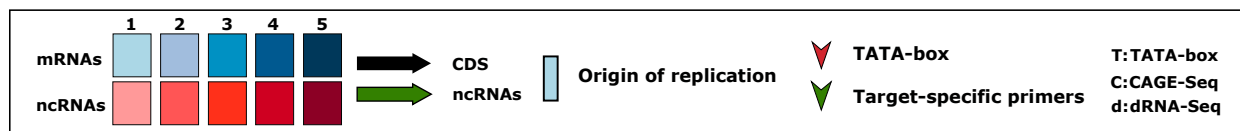

Supplement: Supplementary file 6 — Supplementary Figure 5. [file 41598_2023_43344_MOESM6_ESM.pdf]

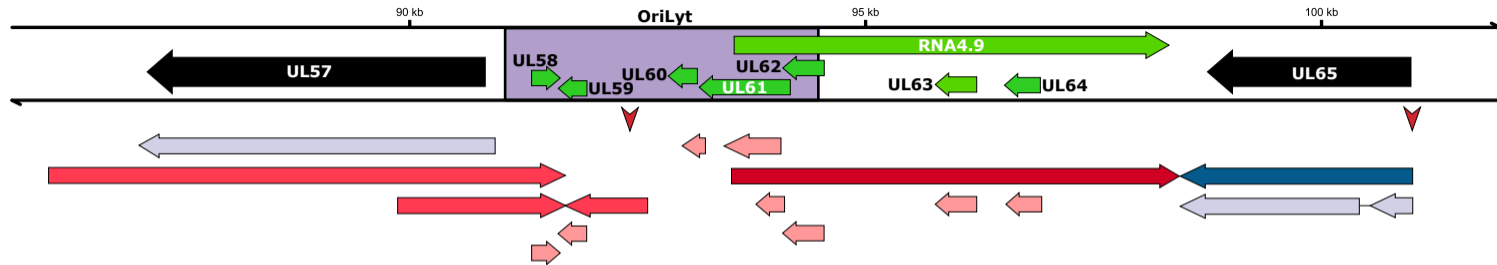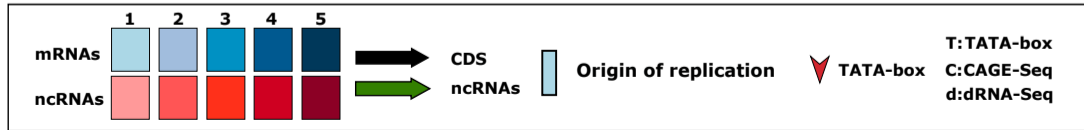

Supplement: Supplementary file 7 — Supplementary Figure 6. [file 41598_2023_43344_MOESM7_ESM.pdf]

38 kb 39 kb 40 kb 41 kb 42 kb 43 kb

**BHLF1**

**OriLyt-L**

**BHRF1**

[illegible]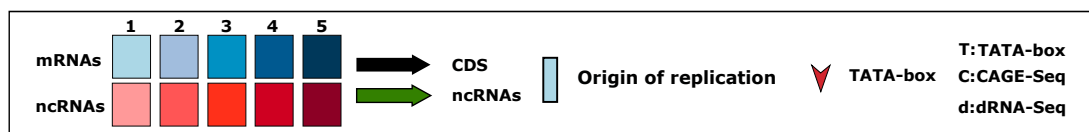

Supplement: Supplementary file 8 — Supplementary Figure 7. [file 41598_2023_43344_MOESM8_ESM.pdf]

**A**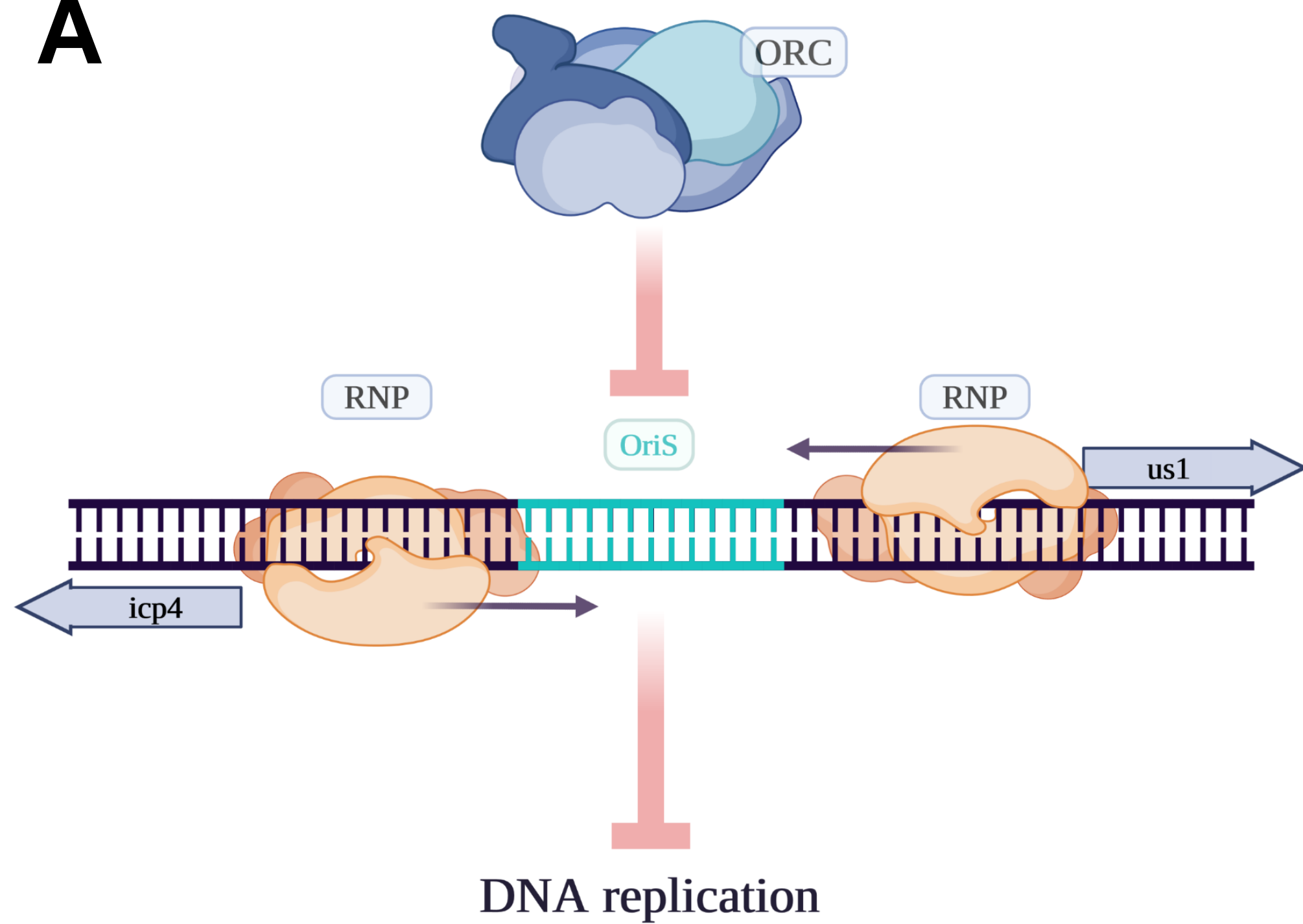**B**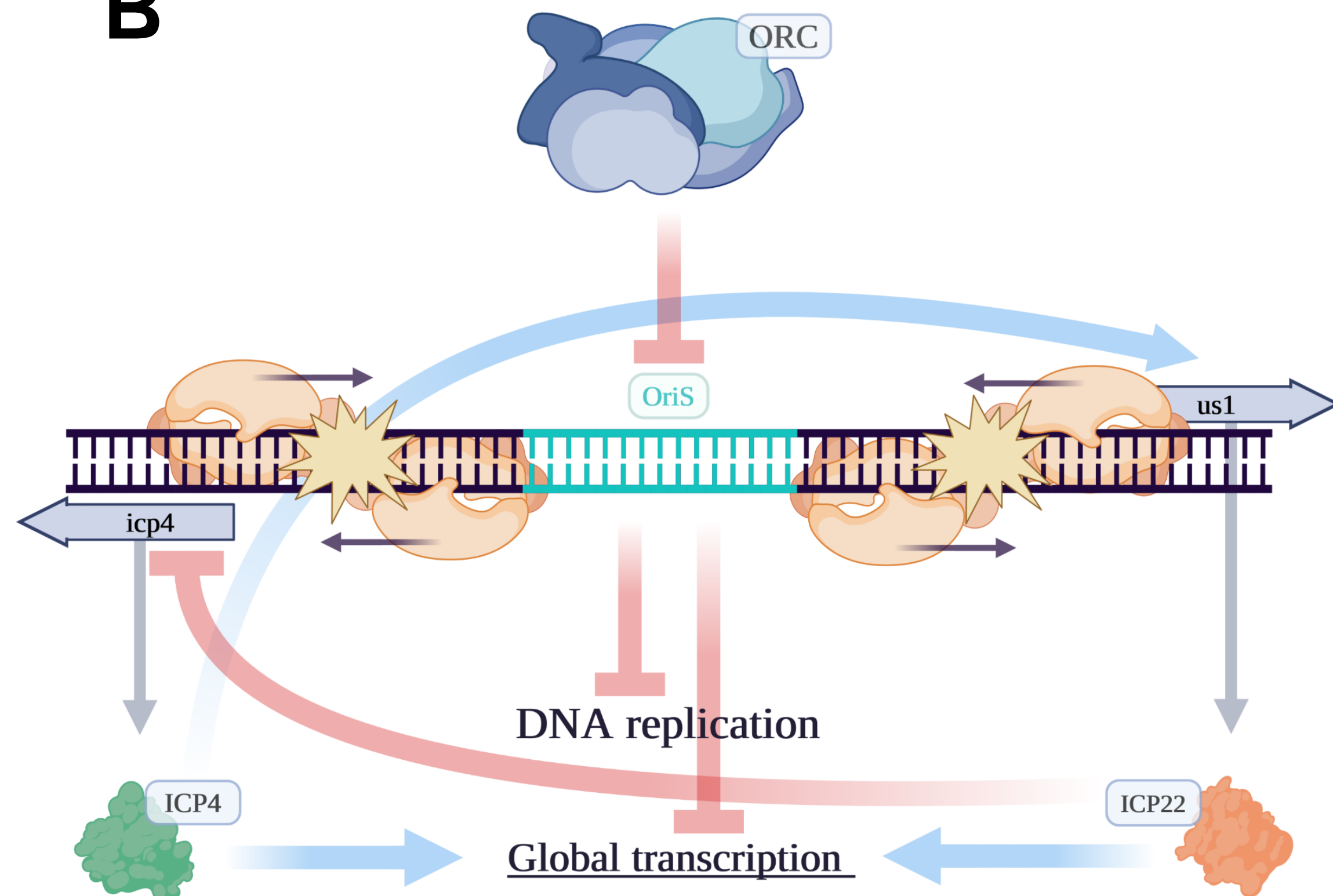**C**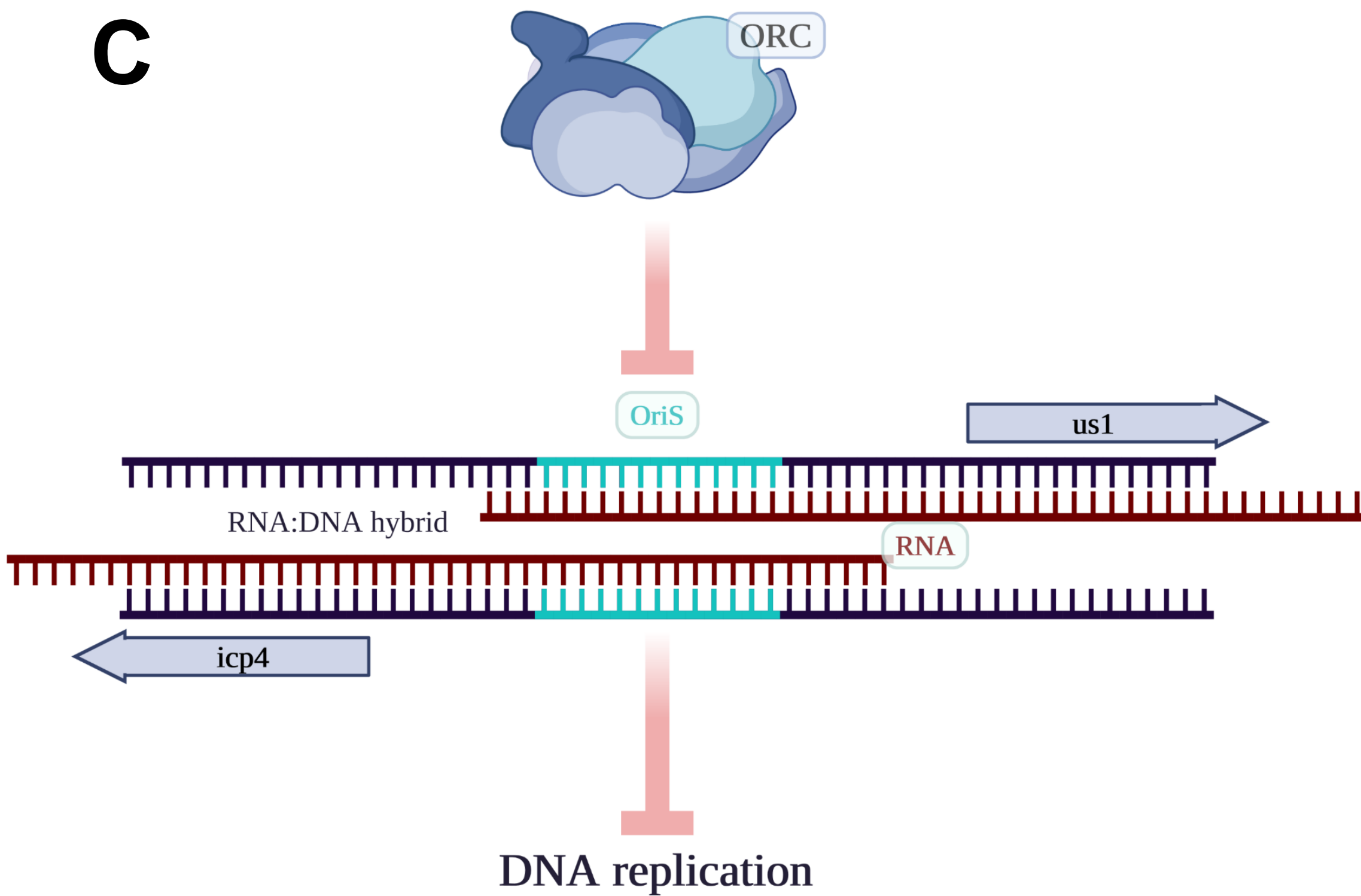**D**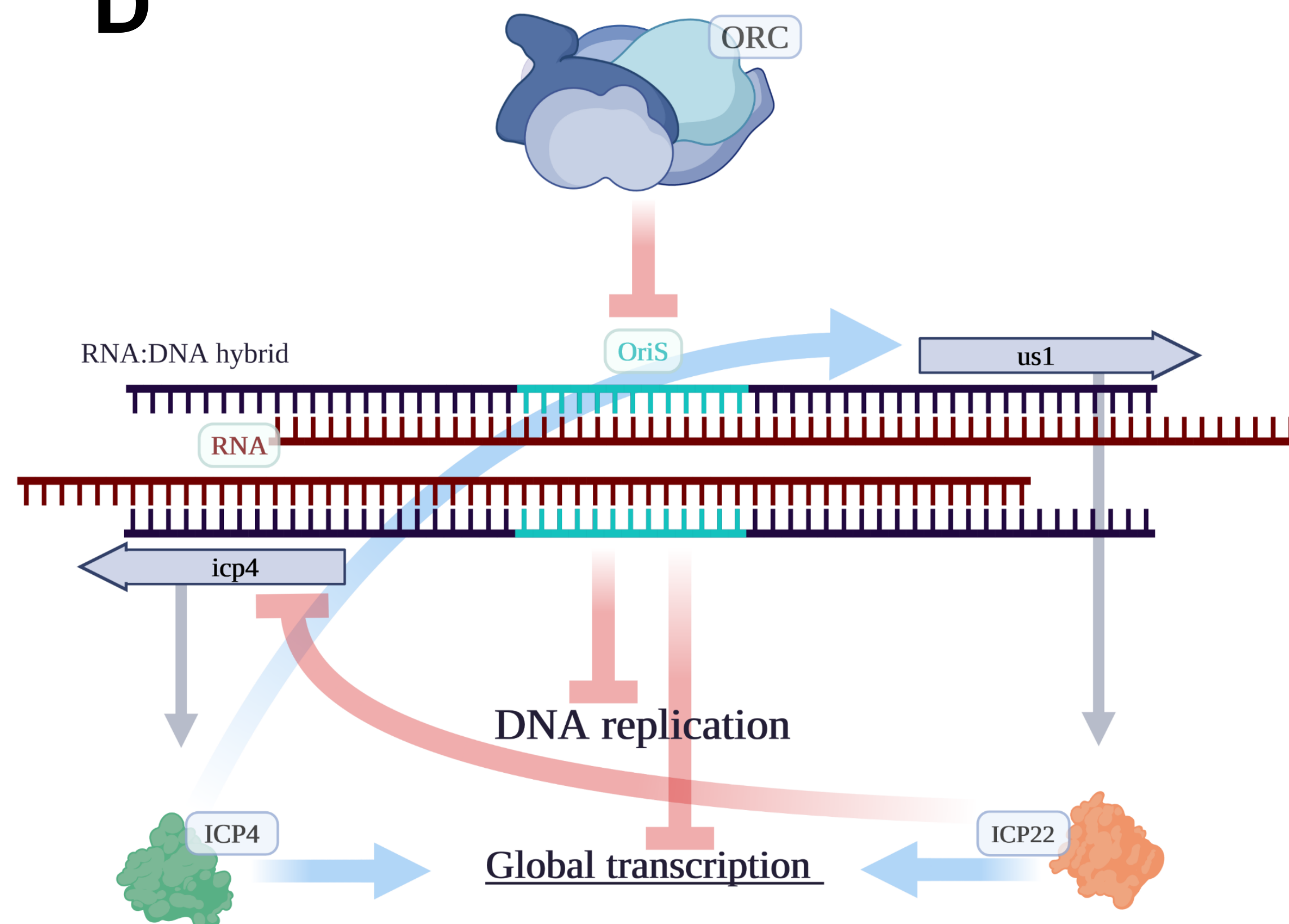

Supplement: Supplementary file 9 — Supplementary Figure 8. [file 41598_2023_43344_MOESM9_ESM.pdf]

**A**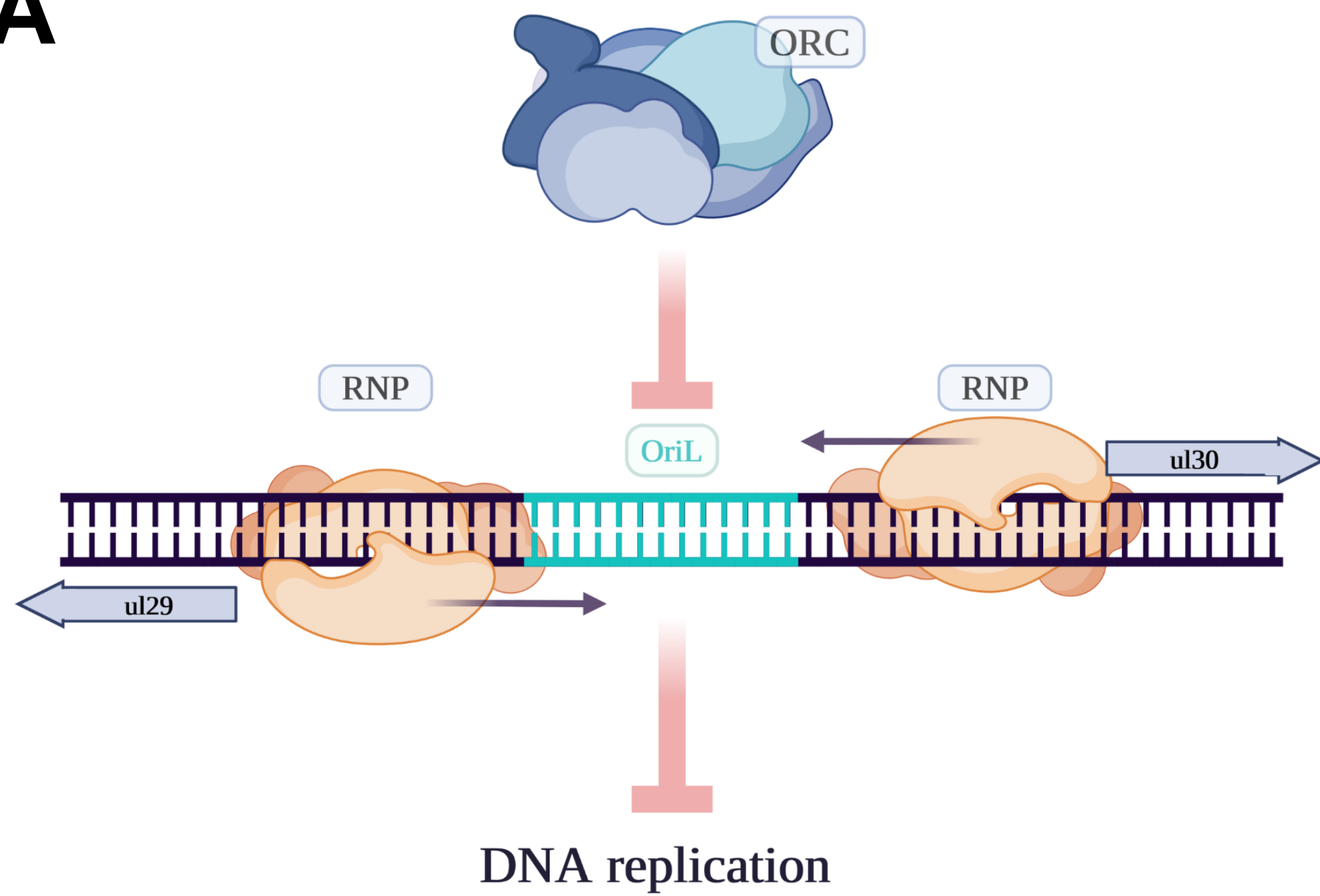**B**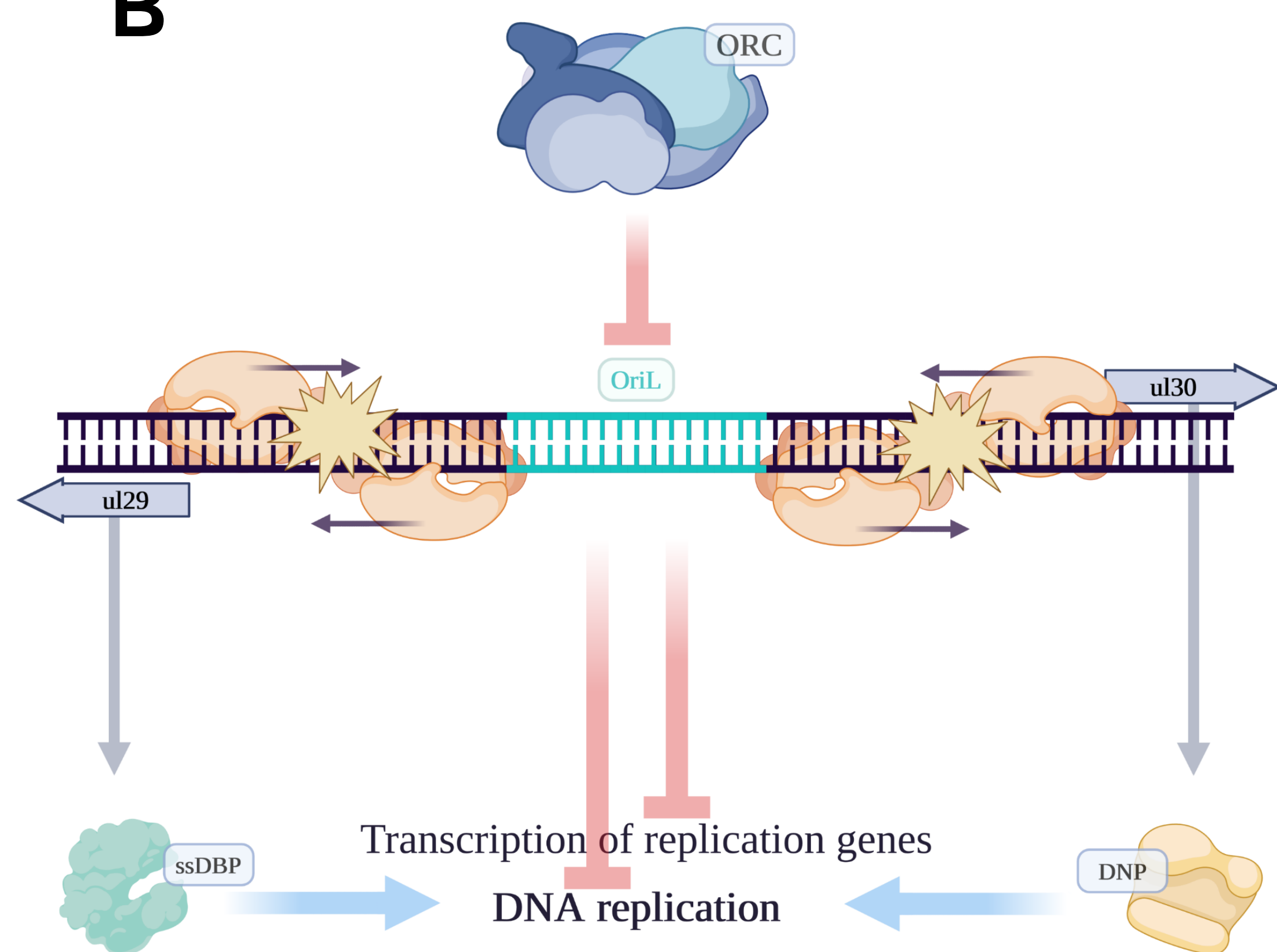**C**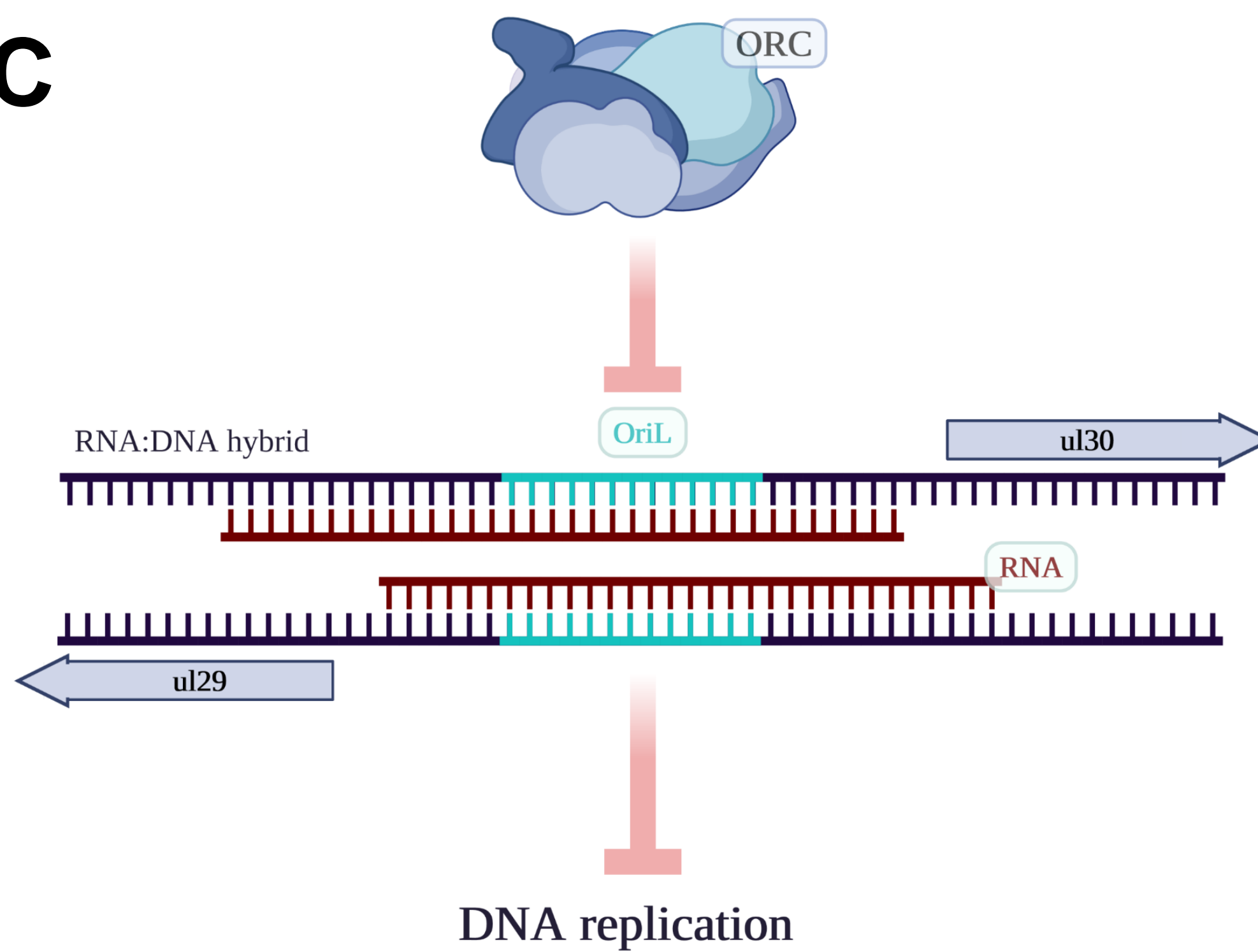**D**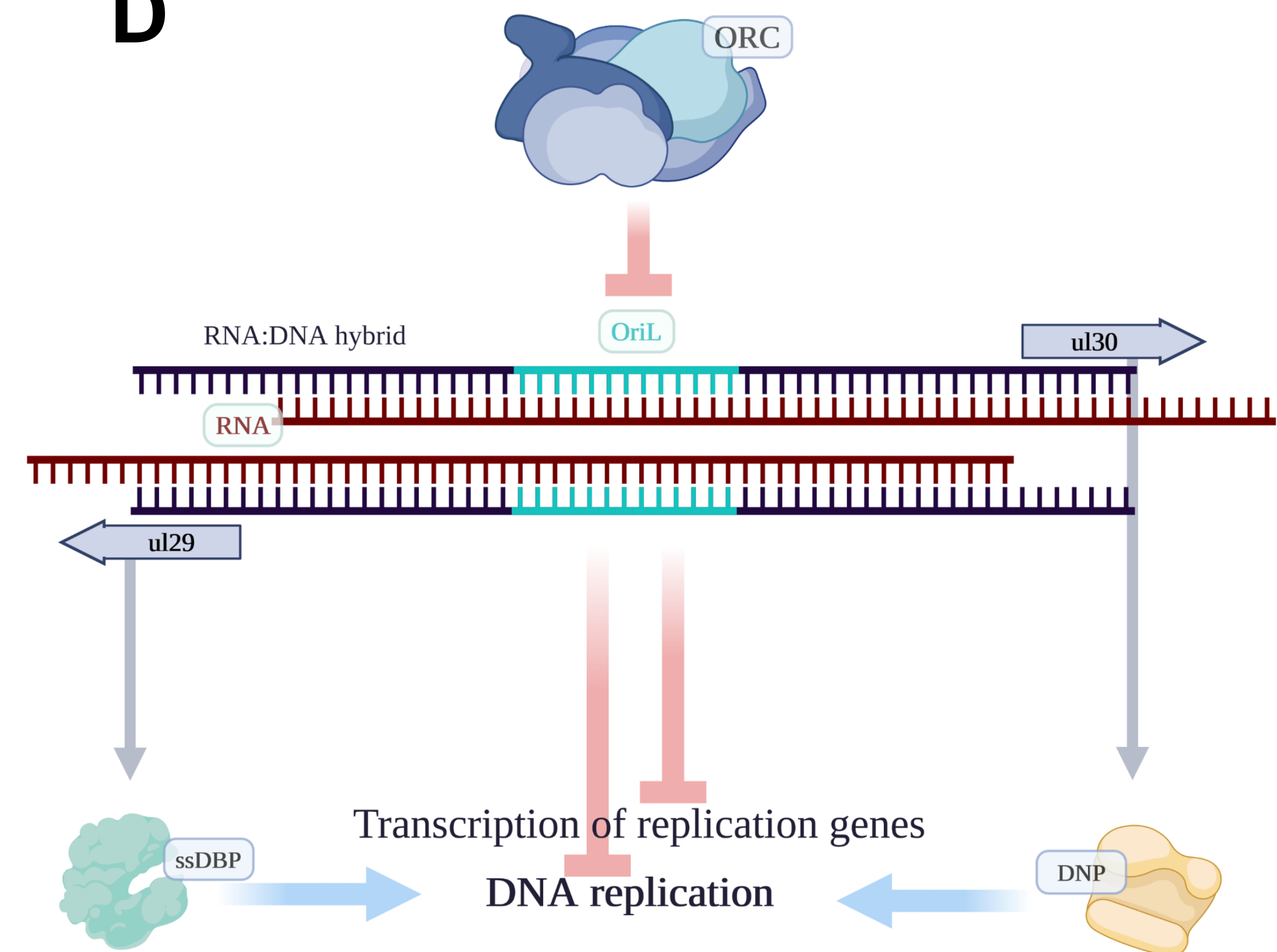

Supplement: Supplementary file 10 — Supplementary Figure 9. [file 41598_2023_43344_MOESM10_ESM.pdf]
