# Supplementary material for: Identification of herpesvirus transcripts from genomic regions around the replication origins
Source: Sci Rep. 2023 Sep 29;13:16395. doi: 10.1038/s41598-023-43344-y (PMC10541914; doi:10.1038/s41598-023-43344-y)
Supplement: Supplementary file 16 — Supplementary Table 6. [file 41598_2023_43344_MOESM16_ESM.docx]

**Supplementary Table 6. Running conditions of qPCR**

|  |  | Temperature | Time | Cycle number |
| --- | --- | --- | --- | --- |
| pre-denaturation |  | 95°C | 15 min | 1 |
| denaturation |  | 94°C | 25 sec | 30 |
| annealing |  | 60°C * | 25 sec |  |
| extension |  | 72°C | 6 sec |  |
