# Supplementary material for: Identification of herpesvirus transcripts from genomic regions around the replication origins
Source: Sci Rep. 2023 Sep 29;13:16395. doi: 10.1038/s41598-023-43344-y (PMC10541914; doi:10.1038/s41598-023-43344-y)
Supplement: Supplementary file 3 — Supplementary Figure 2. [file 41598_2023_43344_MOESM3_ESM.pdf]

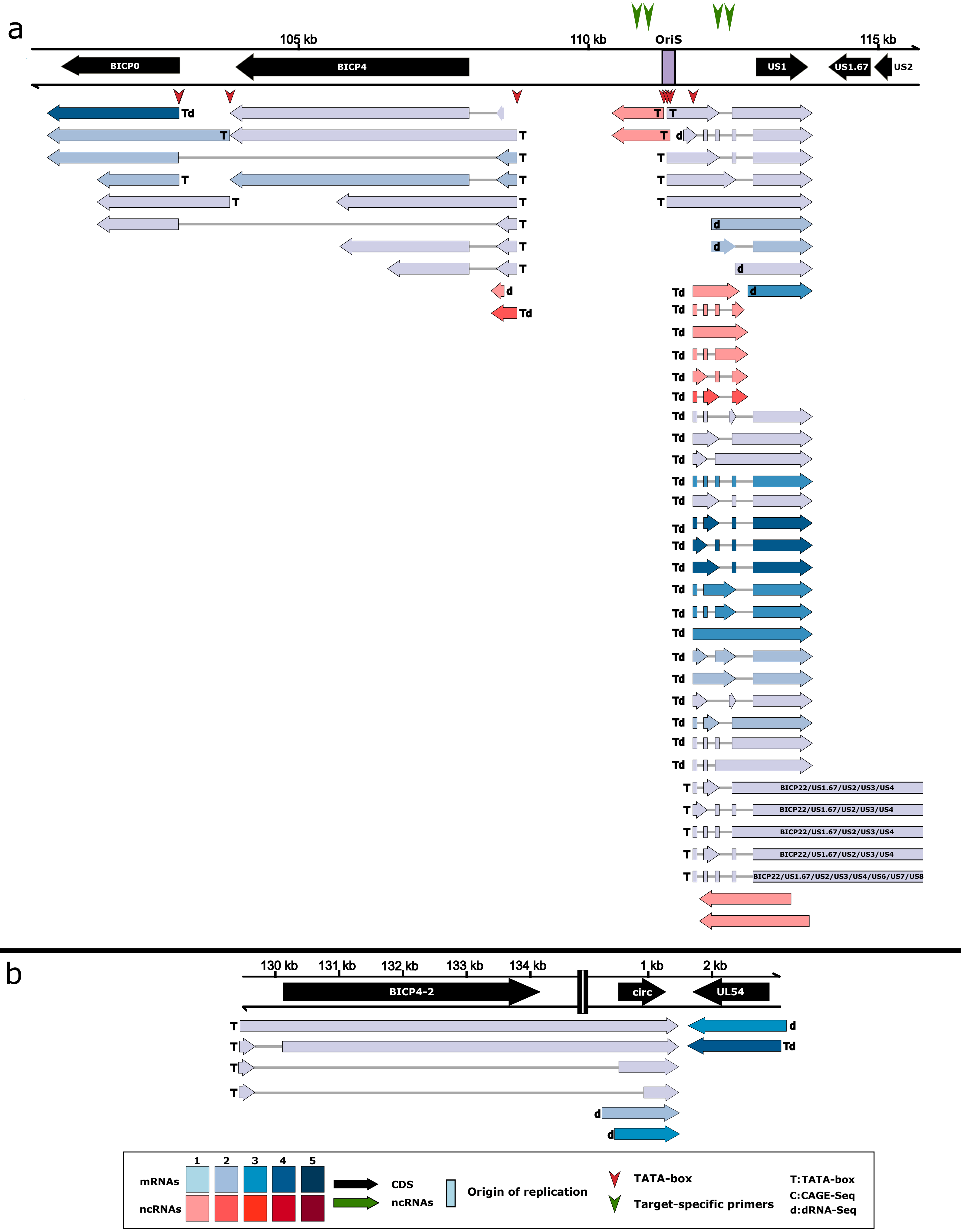
a
105 kb
110 kb
OriS
115 kb
BICP0
BICP4
US1
US1.67
US2
BICP22/US1.67/US2/US3/US4
BICP22/US1.67/US2/US3/US4/US6/US7/US8
b
130 kb
131 kb
132 kb
133 kb
134 kb
1 kb
2 kb
BICP4-2
circ
UL54
mRNAs
ncRNAs
1
2
3
4
5
CDS
ncRNAs
Origin of replication
TATA-box
Target-specific primers
T:TATA-box
C:CAGE-Seq
d:dRNA-Seq
